# Ebola virus-mediated T-lymphocyte depletion is the result of an abortive infection

**DOI:** 10.1371/journal.ppat.1008068

**Published:** 2019-10-24

**Authors:** Patrick Younan, Rodrigo I. Santos, Palaniappan Ramanathan, Mathieu Iampietro, Andrew Nishida, Mukta Dutta, Tatiana Ammosova, Michelle Meyer, Michael G. Katze, Vsevolod L. Popov, Sergei Nekhai, Alexander Bukreyev

**Affiliations:** 1 Department of Pathology, the University of Texas Medical Branch, Galveston, Texas, United States of America; 2 Galveston National Laboratory, the University of Texas Medical Branch, Galveston, Texas, United States of America; 3 Department of Microbiology, University of Washington, Seattle, Washington, United States of America; 4 Department of Medicine, Howard University, Washington, D.C., United States of America; 5 National Primate Research Center, Seattle, Washington, United States of America; 6 Department Microbiology & Immunology, the University of Texas Medical Branch, Galveston, Texas, United States of America; University of Wisconsin-Madison, UNITED STATES

## Abstract

Ebola virus (EBOV) infections are characterized by a pronounced lymphopenia that is highly correlative with fatalities. However, the mechanisms leading to T-cell depletion remain largely unknown. Here, we demonstrate that both viral mRNAs and antigens are detectable in CD4^+^ T cells despite the absence of productive infection. A protein phosphatase 1 inhibitor, 1E7-03, and siRNA-mediated suppression of viral antigens were used to demonstrate *de novo* synthesis of viral RNAs and antigens in CD4^+^ T cells, respectively. Cell-to-cell fusion of permissive Huh7 cells with non-permissive Jurkat T cells impaired productive EBOV infection suggesting the presence of a cellular restriction factor. We determined that viral transcription is partially impaired in the fusion T cells. Lastly, we demonstrate that exposure of T cells to EBOV resulted in autophagy through activation of ER-stress related pathways. These data indicate that exposure of T cells to EBOV results in an abortive infection, which likely contributes to the lymphopenia observed during EBOV infections.

## Author summary

Lymphopenia is a common characteristic of the disease caused by EBOV. We determined that despite the apparent lack of productive infection, EBOV is capable of entering T cells and producing both viral RNAs and proteins. Furthermore, we demonstrate that EBOV causes an abortive infection in T cells due to the presence of a cellular restriction factor. The abortive infection was associated with cell death following ER-stress induced autophagy. Collectively, these findings suggest that abortive infection in T cells is likely to contribute to lymphopenia during Ebola virus disease, which is uniformly linked with the severity of the disease. All EBOV vaccine candidates utilize GP as the sole antigen inducing a protective antibody response and in some clinical trials were shown to induce adverse side effects. The present study suggests that these effects can be associated with GP, which may lead to abortive infection of the vaccine construct in T cells contributing to the inflammatory response to the vaccines.

## Introduction

Ebola virus disease (EVD) is unequivocally one of the most devastating infectious diseases known to exist with previous outbreaks resulting in high fatality rates [1]. Although the 2013–2016 epidemic in West Africa has spurred the development of candidate vaccines and therapeutics, supportive care remains the primary course of treatment for EVD [2–4]. The particularly virulent nature of Ebola virus (EBOV) stems from its ability to rapidly disarm the host’s immune system by targeting antigen-presenting cells, including macrophages and dendritic cells and thus inhibiting the development of a virus-specific adaptive immune response [5–7].

Although T cells themselves remain refractory to productive viral infection, lymphopenia is a common characteristic of EVD [5, 8–15]. The extent of lymphopenia is one of the strongest correlates of EVD outcome: the ability of patients to maintain T cells over the course of infection is observed in survivors of EVD whereas the opposite is nearly universally observed in fatalities [16, 17]. Studies of the immune status of EVD patients during the 2013–2016 EBOV epidemic in West Africa demonstrated that survivors develop a robust EBOV-specific cell-mediated response, which in some cases persists beyond viral clearance [18–20]. Accordingly, studies with the mouse model demonstrated the protective role of CD8^+^ and CD4^+^ T cells in EVD [21, 22]. Gene expression studies demonstrated that survival was associated with low expression of CTLA-4, PD-1 and PD-L1 and low diversity of T cell receptors, as compared to fatal cases [23, 24]. Furthermore, EVD survivors demonstrated lower enrichment of genes associated with interferon signaling in blood compared to patients with fatal infection [25]. These data are consistent with the findings using a monkey model of EVD, that a continuous type I interferon response is likely to inhibit the ability of lymphocytes to contribute the protection [26]. Increased expression of the death ligand, FasL, and the apoptosis inducer, TRAIL, have been shown in *in vitro* models of disease [17, 27]. The massive burst in inflammatory mediator production, which is also known as the cytokine storm, is also a likely contributor to the extent of lymphopenia observed *in vivo* [17, 28]. Though numerous pathways have been associated with EBOV-induced lymphopenia [7], little is known about the mechanisms leading to this condition.

Recently, we demonstrated that EBOV directly binds and stimulates CD4^+^ T cells via interactions between virion-associated phosphatidylserine (PS) and the cellular receptor T-cell immunoglobulin mucin receptor 1 (Tim-1) [29]. Direct stimulation of T cells following exposure to EBOV was shown to elicit the production of inflammatory mediators, which contributed to cell death *in vitro* and likely contributed to the cytokine storm and lymphopenia observed *in vivo* [9, 29]. Based on our previous findings, we further investigated the significance of direct exposure of EBOV to isolated primary human CD4^+^ T cells and the Jurkat T cell line. Using multiple known stages of viral replication as a basis for analysis, we investigated whether EBOV could directly bind and infect T cells in the absence of productive viral replication. We determined that EBOV causes an abortive infection in T cells, which ultimately leads to ER-stress induced autophagy and subsequent cell death.

## Results

### Detection of viral RNAs in CD4^+^ T cells

**Fig 1:**
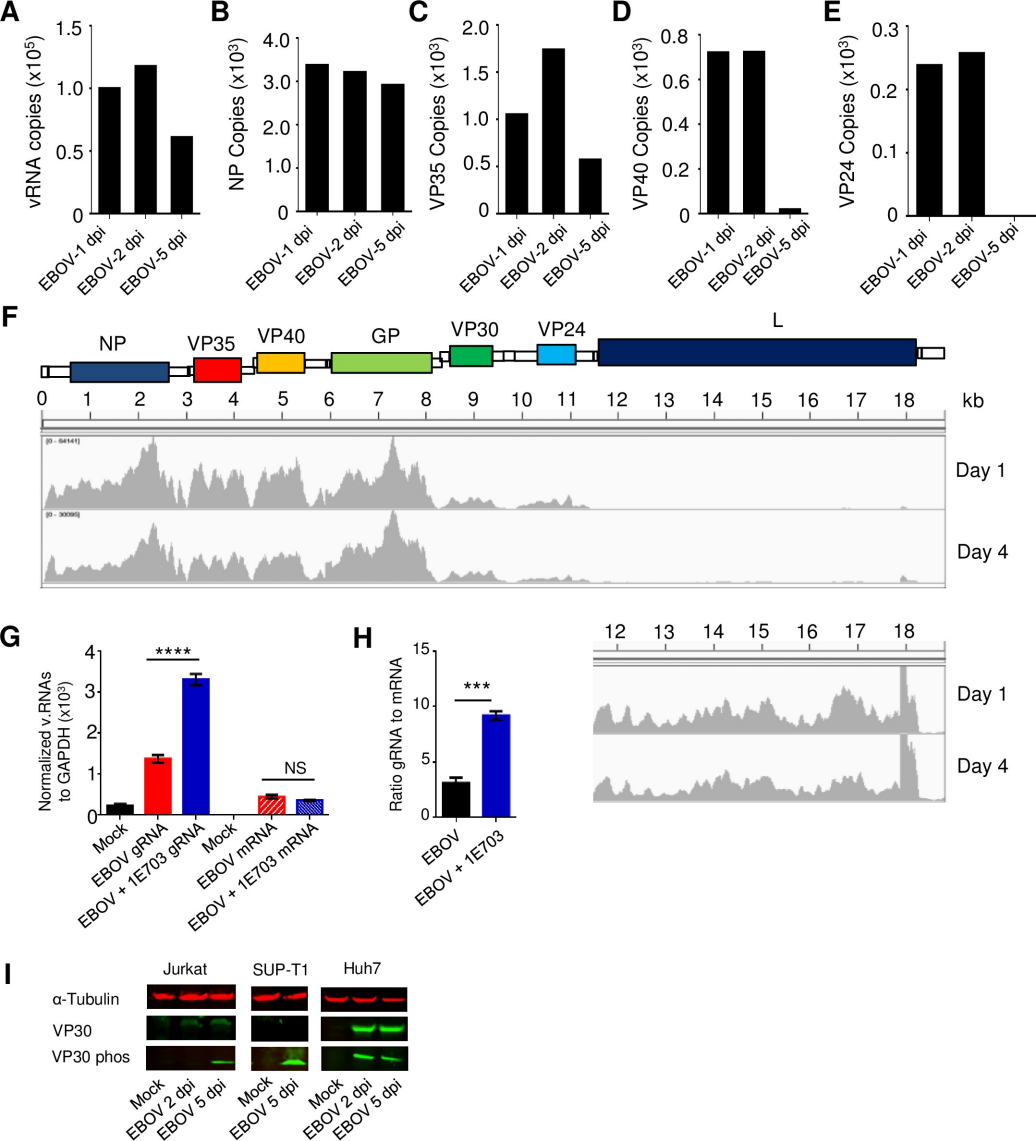
Abortive EBOV infection in CD4^+^ T-cells. (**A-E**) The number of viral genomic RNA (A) and specific viral mRNAs (B-E) copies/ng in CD4^+^ T-cells exposed to EBOV for 1, 2 and 5 days, determined by DDRT-PCR with background signals in mock-infected cells subtracted. (**F**) Histograms displaying the relative number of sequence reads for each viral mRNA at day 1 and 4 as determined by deep sequencing. Bottom right: the zoomed in fragment of the reads corresponding to the 5’-terminal part of the genome, which includes the L gene. (**G**) Numbers of viral genomic RNA (gRNA, left) and mRNA (right) copies normalized to GAPDH in mock-infected, EBOV-infected and 1E7-03 treated EBOV-infected cells. (**H**) Ratios of viral gRNA to mRNA in non-treated and 1E7-03 treated cells. (**I**). Analysis of the total and phosphorylated VP30 in Jurkat and SUP-T1 T cells and Huh7 cells incubated with EBOV for 2 or 5 days at MOI 3 PFU/cell by Western blot. Panel A: 1 of 3 independent experiments. Panels B-E: Average of duplicate reads from EBOV-exposed CD4^+^ T-cells isolated from one of 3 donors. Panel F: Representative number of reads from deep sequencing results depicted as histogram shown from 1 of 4 independent donors. Panel G: data from triplicate samples from one representative donor; two donors were analyzed resulting in similar data. *** P<0.001, **** P<0.0001.

We previously demonstrated that EBOV enters CD4^+^ T cells using a trypsin digestion assay and by confocal microscopy [29]. We next sought to determine whether the viral RNA polymerase complex is functional in CD4^+^ T cells and Jurkat T cells cultured with EBOV by measuring the relative genomic RNA (gRNA) and protein coding messenger RNA (mRNA) at multiple time points by strand-specific quantitative droplet digital RT-PCR (DDRT-PCR). Viral gRNA levels were detectable in CD4^+^ T cells on days 1 and 2 post infection at approximately 1x10^5^ copies/mg followed by some decrease at day 5 (**Fig 1A**); comparable levels of gRNA were detected in Jurkat cells (**S1 Fig).** Viral mRNA was detected in both CD4^+^ T cells and the Jurkat cells cultured with EBOV (**Fig 1B–1E and S1 Fig)**. We noted that the levels of detected viral mRNA correlated with their decreased transcription from the 3’ end of the viral gRNA. For example, NP mRNA, which is transcribed from the gene located at the 3’ end of the genome, was one log higher than VP24, whose gene is located closer to the 5’ end of the genome. This finding was verified by deep sequencing analysis of CD4^+^ T cells exposed to EBOV from 4 independent donors (**Fig 1F**).

We next used the inhibitor of protein phosphatase 1 (PP1) 1E7-03, which was previously shown to block dephosphorylation of the EBOV transcription factor VP30 and inhibit viral growth [30], to determine if the observed RNAs were the result of *de novo* synthesis. VP30 supports transcription only in a dephosphorylated state; its phosphorylation results in the block of transcription and switch of the EBOV polymerase activity to replication [30–32]. Hence, blocking dephosphorylation of VP30 was expected to increase replication of the EBOV genome and increase levels of viral genomic and complementary RNAs. Consistent with this prediction, we noted a significant increase in viral genomic/complementary RNA in EBOV-exposed CD4^+^ T cells treated with 1E7-03 in comparison to untreated, EBOV-exposed CD4^+^ T cells (**Fig 1G**). Furthermore, in the presence of the inhibitor, mRNA synthesis remained slightly lower and the ratio of EBOV genomic RNAs to mRNAs was significantly higher than non-treated cells (**Fig 1H**). Interestingly, analysis of Jurkat and SUP-T1 human T cell lines demonstrated much greater levels of phosphorylated VP30 compared to Huh7 hepatocellular carcinoma cells incubated with EBOV for 2 or 5 days (**Fig 1I**), suggesting a mechanism for the reduced viral replication in lymphocytes. Overall, these findings indicate that replication of viral RNAs occurs in EBOV-exposed lymphocytes.

### EBOV entry into CD4^+^ T cells results in *de novo* synthesis of viral proteins

**Fig 2:**
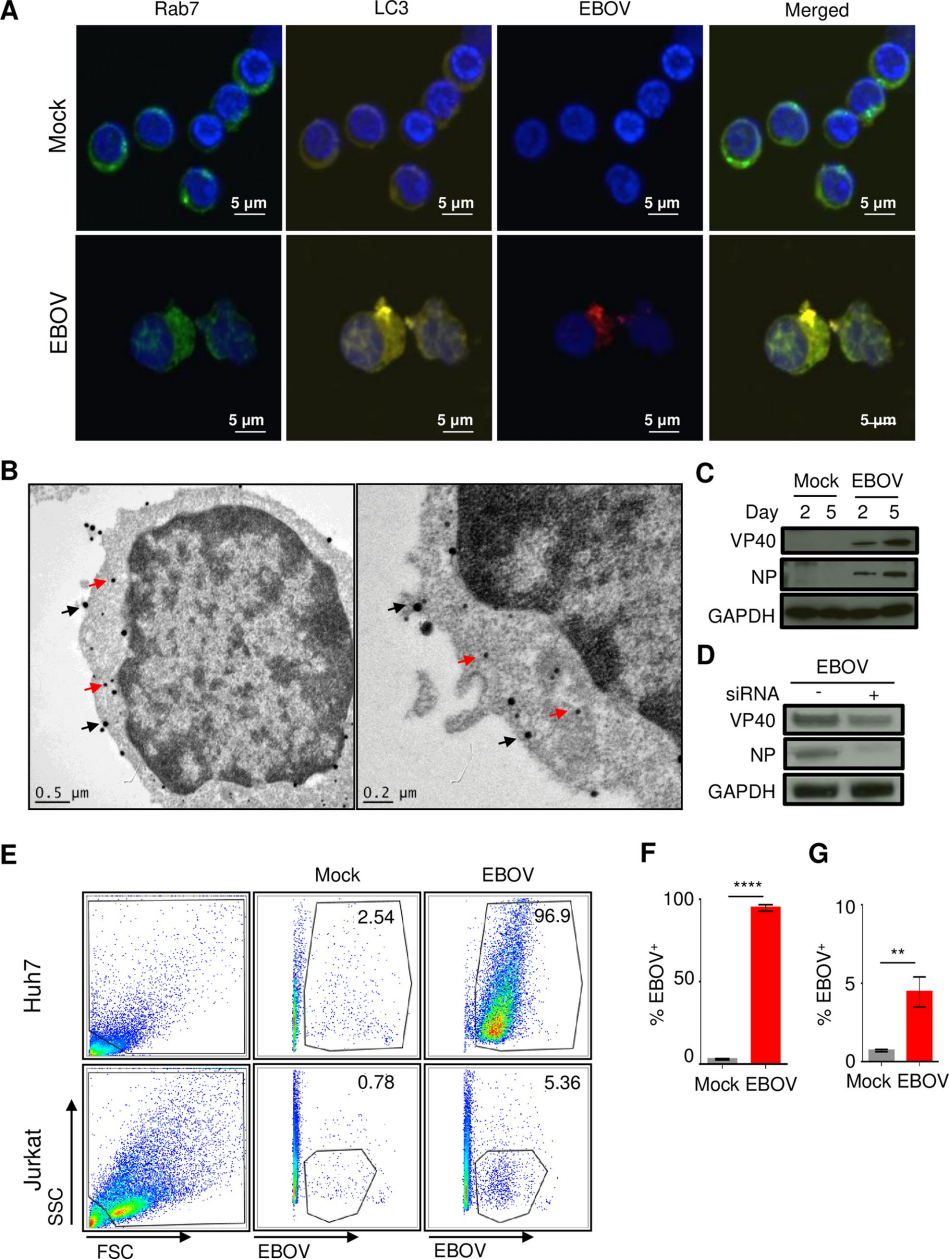
EBOV viral proteins are readily detectable in CD4^+^ T-cells. (**A**) Confocal microscopy analysis of EBOV-exposed CD4^+^ T-cells for colocalization of the late-endosomal marker Rab7 (green), the lysosomal marker, LC3 (yellow) and EBOV antigens (red); nuclei are stained with DAPI (blue). (**B**) Dual immuno-gold labeling TEM of CD4^+^ T-cells exposed to EBOV: immunostained for CD3 with ~40 nm gold particles (black arrows) and for EBOV antigens with ~15 nm gold particles (red arrows). (**C**) Western blot analysis of EBOV VP40 and NP proteins in CD4^+^ T-cells incubated with EBOV for 2 or 5 days. (D) Western blot analysis of EBOV VP40 and NP proteins in EBOV-exposed Jurkat cells transfected with siRNAs targeting VP40 and NP. (**E**) Flow cytometry analysis of Jurkat cells and Huh7 cells incubated with EBOV. (**F,G**) Percentages of EBOV-positive Huh7 (F) and Jurkat (G) cells determined by flow cytometry, mean values based on triplicate samples ±SE. ** P<0.01, **** P<0.0001 (Student’s t-test). A, B: representative images from one of three independent experiments. C, D: representative images from one of two independent experiments. E–G: representative of one of two independent experiments performed.

As our previous studies provided evidence regarding the entry of EBOV virions in T cells, we assessed whether EBOV co-localized with markers of intracellular organelles specific for late-endosomes (Rab7) and autophagosomes (LC3) soon after exposure to EBOV. Using confocal microscopy analysis with antibodies raised against EBOV virus-like particles (VLPs), which contain the NP, VP40 and GP proteins, we determined that EBOV was readily detectable in both organelles within 2 hours of the addition of EBOV to T cells at a MOI of 3 PFU/cell (**Fig 2A**); however, extensive co-localization was primarily observed in organelles staining positive for autophagosome marker LC3. These findings suggest that a portion of internalized EBOV virions are directly targeted to autophagosomes shortly upon entry.

We next determined if viral proteins were expressed in CD4^+^ T cells using multiple approaches. Isolated CD4^+^ T cells were cultured in the presence of EBOV at a MOI of 1 PFU/cell for 48 hours and analyzed by transmission electron microscopy (TEM) with antibodies raised against EBOV VLPs. We used dual immunogold labeling, which revealed that cells staining positive for CD3 (40 nm gold particles) also stained positive for viral antigens (15 nm gold particles) (**Fig 2B** and **S2 Fig**). To further corroborate protein expression in CD4^+^ T cells, we conducted Western blot analysis on samples collected at days 2 and 5 post exposure to EBOV. The EBOV VP40 and NP were readily detectable, and increase in levels was observed from day 2 to day 5, which is suggestive of *de novo* synthesis (**Fig 2C**).

To confirm *de novo* synthesis, Jurkat T cells were mock-transfected or transfected with siRNAs targeting EBOV VP40 and NP using the Neon electroporation system. As shown in **Fig 2D**, EBOV-infected cells transfected with siRNAs expressed markedly lower levels of both viral proteins. Lastly, intracellular staining of Jurkat cells co-cultured with EBOV (MOI of 3 PFU/cell) for 48 hours using antibodies raised against EBOV VLPs revealed a distinct population of cells positive for viral antigens (**Fig 2E**). Comparatively, 96% of Huh7 cells were positive for EBOV antigens whereas only ~5% of Jurkat cells were positive for EBOV antigens further suggesting that although viral synthesis occurs in CD4^+^ T cells, the rate of infection and/or protein synthesis is significantly lower (**Fig 2F and 2G**). These findings were further confirmed by confocal microscopy, which similarly showed that the percentage of Jurkat T cells expressing viral antigens following their cultivation with EBOV (MOI of 3 PFU/cell) for 48 hours is markedly lower than that observed in Huh7 cells (**S3A and S3B Fig**). Furthermore, neither Jurkat cells (**S3 Fig**) nor CD4^+^ T cells (**Fig 2A**) demonstrated the presence of inclusion bodies in the cytoplasm, which were observed in Huh7 cells (**S3 Fig**). Exposure of Jurkat T cells (MOI of 3 PFU/cell) to recombinant EBOV expressing GFP (EBOV-GFP) [33] for 1 hour, followed by a wash and incubation for 48 hours provided further evidence of *de novo* synthesis as GFP expression was clearly detectable (**S4A and S4B Fig**). Despite the detection of viral proteins, no infectious virus was detected in cell-free supernatant collected from EBOV-GFP exposed Jurkat T cells (**S4C and S4D Fig**).

### A cellular restriction factor blocks replication of EBOV particles in CD4^+^ T cells

Previous studies have shown that specific cell types express cellular restriction factors that block viral replication. The fusion of cells permissive to viral infection with non-permissive cell lines have led to the discovery of intrinsic antiviral factors and to the discovery of novel viral evasion strategies [34]. We therefore conducted similar experiments to determine if T cells express a cellular restriction factor or if they are missing a cellular factor needed for viral replication. The permissive hepatic cell line, Huh7, is readily infected with EBOV, whereas Jurkat T cells, have been shown to be non-permissive to EBOV-infection. We therefore combined DiL labelled Jurkat T cells with DiD labelled Huh7 cells and fused the cells with Clonal Cell-HY-PEG (Stem Cell Technologies).

**Fig 3:**
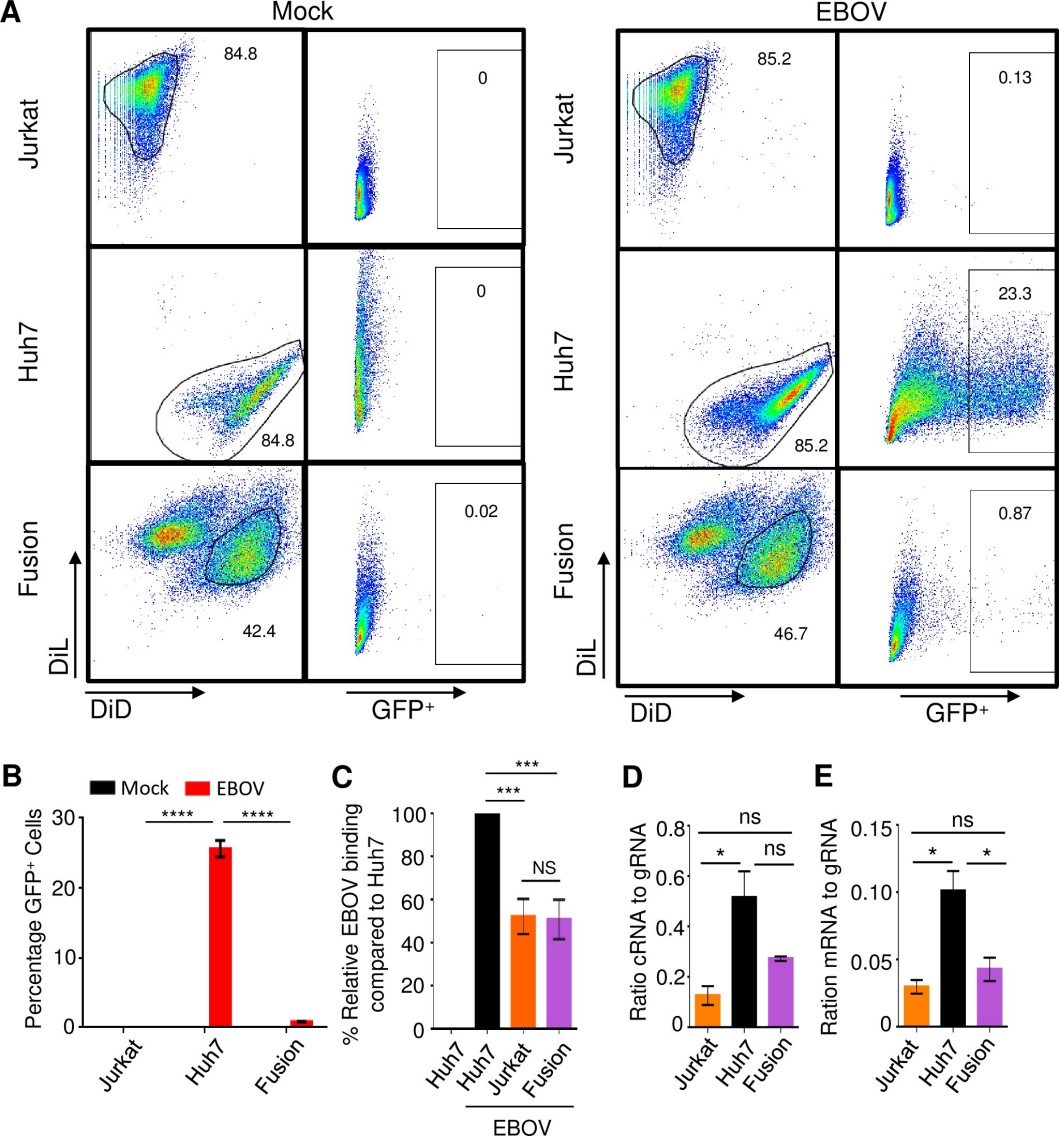
CD4^+^ T-cells express a cellular restriction factor that blocks EBOV replication. (**A**) Flow cytometry analysis of fusion cells generated from DiL-labeled Jurkat cells and DiD-labeled Huh7 cells infected with EBOV-GFP. (**B**) Mean percentages of GFP^+^ cells based on triplicate samples ±SE. (**C**) Mean relative binding of EBOV based on triplicate samples ±SE. (**D**) Ratio cRNA to gRNA based on triplicate samples ±SE. (**E**) Ratio mRNA to gRNA based on triplicate samples ±SE. B: representative of one of 3 independent experiments. C, D, E: representative of one of two independent experiments. * P<0.05; *** P<0.001; **** P<0.0001; n.s., not significant (Student’s t-test).

Jurkat cells (DiL labelled), Huh7 (DiD labelled) and the Jurkat-Huh7 fusion cell line were exposed to EBOV-GFP at an MOI of 0.3 PFU/cell for 3 days; the low MOI was selected due to the concerns of the reduced stability of the fusion cells. The cells were then harvested, fixed and analyzed by flow cytometry for GFP expression. As shown in **Fig 3A**, the relative expression of GFP in Jurkat cells was minimal (0.13%) whereas 23.3% of Huh7 cells were GFP^+^ at day 3. Interestingly, gating of the double positive (DiL^+^DiD^+^) fusion cells revealed that only 0.87% of cells were GFP^+^ at day 3, demonstrating that the fusion of Jurkat cells with Huh7 cells resulted in a statistically significant 26.8-fold reduction in GFP^+^ cells as compared to Huh7 cells (**Fig 3B**). This finding indicates that T cells express a cellular restriction factor as opposed to lacking a cellular factor required for replication of EBOV particles.

To determine if viral attachment was the primary cause of the reduction in GFP^+^ cells, we utilized a previously developed binding assay in which various cell types are incubated with EBOV added at MOI of 3 PFU/cell on ice for 2 hours, washed, stained and analyzed by flow cytometry. Using Huh7 cells as a control, we determined that binding of EBOV to the fusion cell line was reduced to approximately 50% of that observed in Huh7 cells, which was similar to the level observed in Jurkat cells (**Fig 3C**). This finding suggests that while attachment is reduced in the fusion cell line, it does not account for the 26.8-fold reduction in GFP as attachment is only reduced by half.

We next conducted a thorough investigation of the relative levels of viral RNAs within Jurkat T cells, Huh7 cells and the fusion cell line. Using a series of primer sets, we quantified the relative ratio of viral gRNA, complementary (antigenomic) RNA (cRNA) and mRNA in cells infected with EBOV at a MOI of 0.3 PFU/cell for 72 hours. The ratio of cRNA to gRNA in the fusion cell line was reduced in comparison to Huh7 cells (**Fig 3D**), but the reduction did not reach statistical significance, while the ratio of mRNA to gRNA was significantly reduced by 59% (**Fig 3E**). These data are consistent with the greater phosphorylation of EBOV transcription factor VP30 in lymphocytic cell lines (**Fig 1I**).

**Fig 4:**
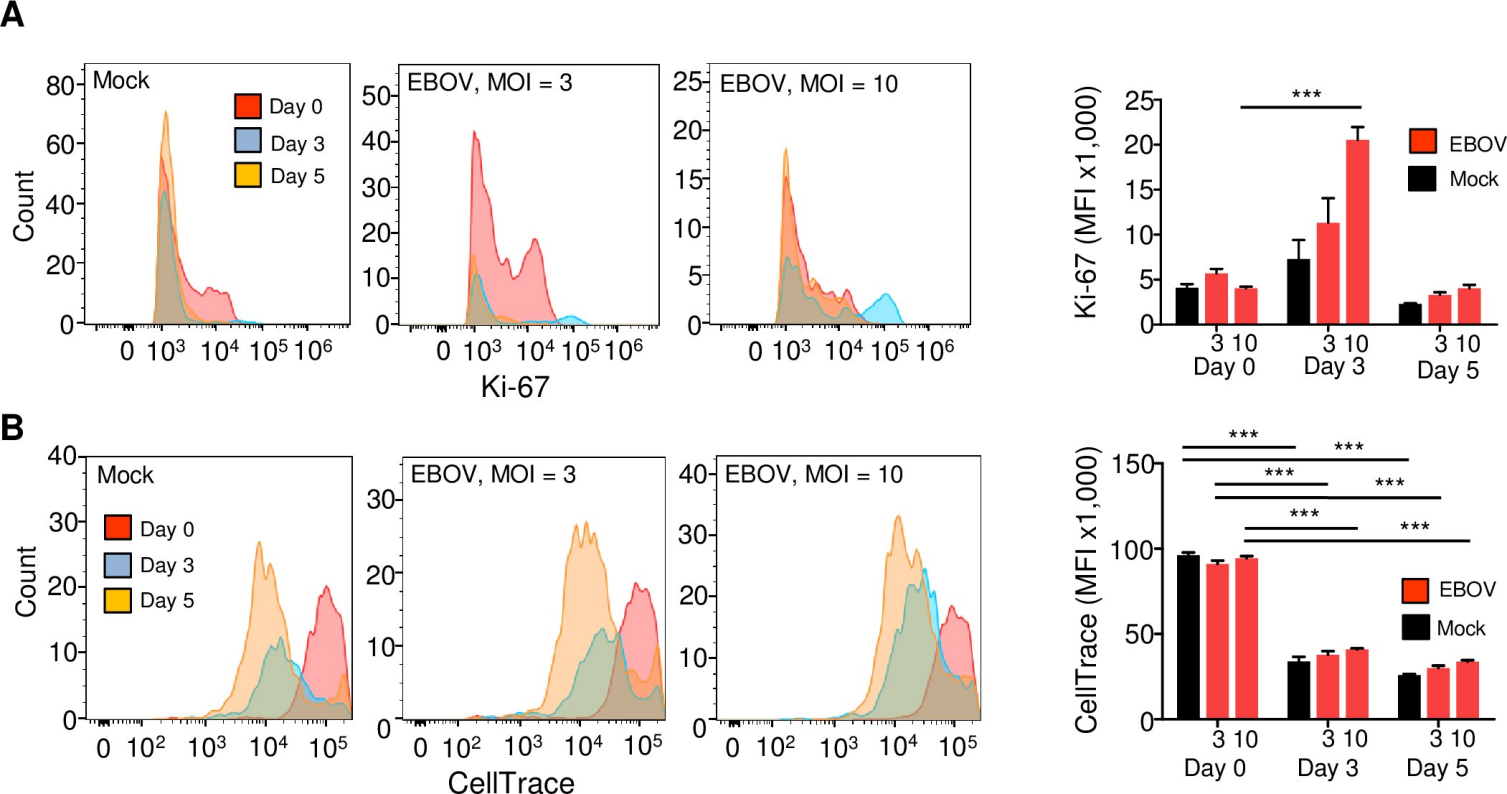
The limited EBOV replication in CD4^+^ T-cells is not related to the anergic state. Flow cytometry analysis of CD4^+^ T cells from a representative donor exposed to EBOV at a MOI of 3 or 10 PFU/cell for 3 or 5 days. Cells were stained, and viable cell populations negative for LIVE/DEAD Fixable Aqua Dead Cell Stain were gated and analyzed for Ki-67 and for cell division. Left: representative primary flow cytometry data. Right: mean values based on triplicate samples ±SE, the MOI is indicated under the bars. (**A**) Analysis of for the activation marker Ki-67. (**B**) Analysis of proliferation by pre-staining of cells with the permanent dye CellTrace. Statistical differences compared to the day 0 values based on triplicate samples: *** P<0.001 (Student’s t-test).

To exclude the alternative possibility that the limited EBOV replication in T cells is related to the anergic state or a block in proliferation, we stained primary human CD4^+^ T cells with the permanent dye CellTrace Far-Red and incubated the cells with EBOV at a MOI of 3 or 10 PFU/cell. On days 3 and 5, cells were stained and viable cells were analyzed by flow cytometry for the activation marker Ki-67 and for cell division by the dilution of CellTrace. We found a sharp increase in the levels of Ki-67 on day 3, followed by the reduction to baseline levels on day 5 (**Fig 4A**). We also found a continuous division of cells on both days 3 and 5 (**Fig 4B**). These data suggest that EBOV-exposed T cells are capable of proliferation and do not enter the anergic state.

### Abortive infection of CD4^+^ T cells results in ER stress-induced autophagy

**Fig 5:**
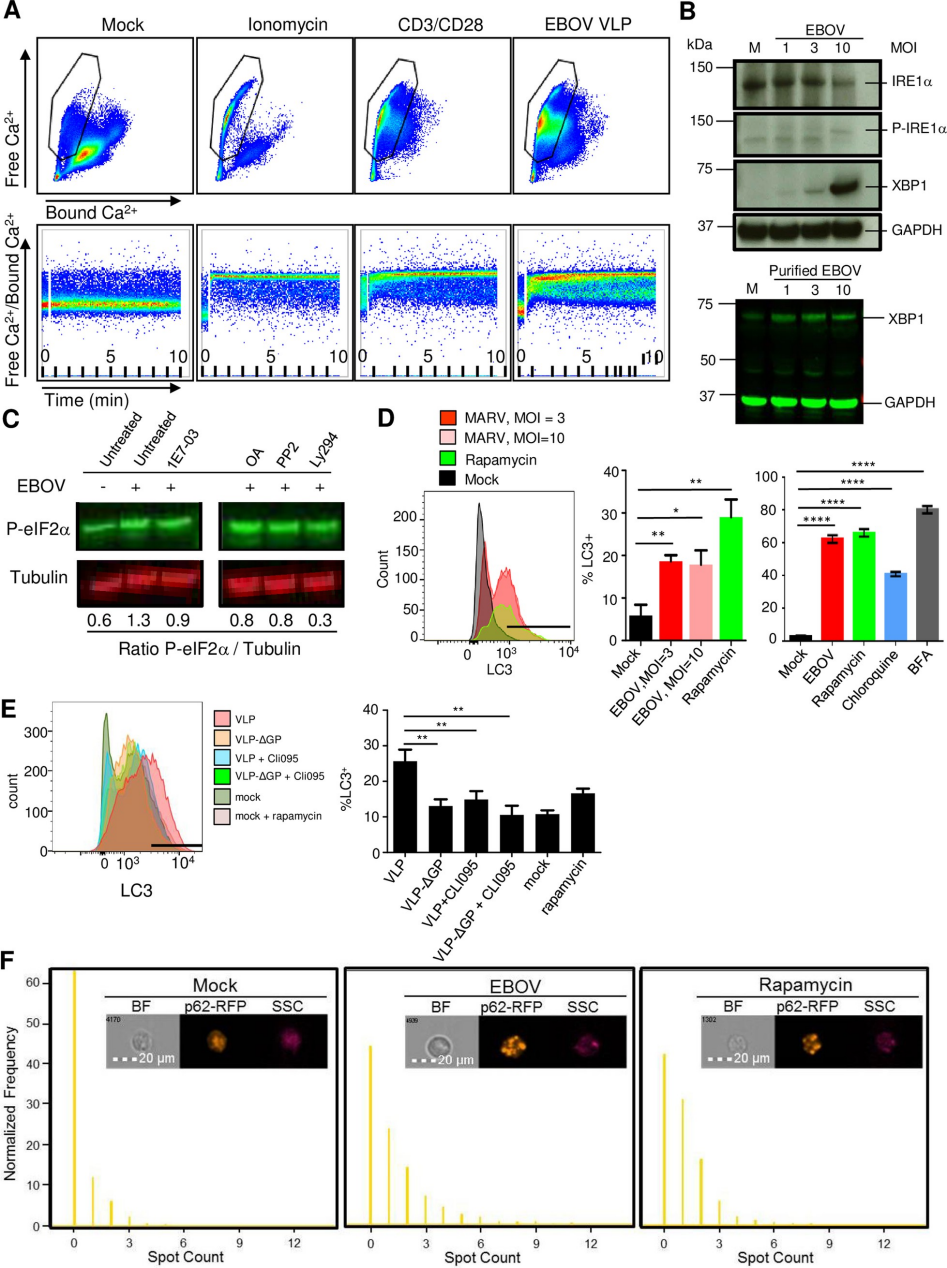
Abortive infection of T-cells results in ER-stress induced autophagy. (**A**) Flow cytometry analysis of intracellular Ca^2+^ levels in primary CD4^+^ T-cells exposed to ionomycin, CD3/CD28 beads or EBOV VLPs for 10 min. Top dot plots results represent bound Ca^2+^ (X axis) and free Ca^2+^ (Y axis). Bottom dot plots represent the ratio of free Ca^2+^/Bound Ca^2+^ over time during the 10 min stimulation. (**B**) Top: Western blot analysis of isolated CD4^+^ T-cells exposed to EBOV at various MOIs or mock-infected cells (M) for markers of ER-stress phospho-IRE1a and XBP1, as well as dephosphorylated form of IRE1a. Bottom: Western blot analysis of isolated CD4^+^ T-cells exposed to purified EBOV for XBP1. (**C**) Western blot analysis of phosphorylated eIF2a (P-eIF2a) in CD4^+^ T-cells exposed to EBOV in the presence or absence 1E7-03, okadaic acid (OA), a Lck inhibitor PP2 or a PI3K inhibitor Ly294. The ratio of phospho-eIF2a to tubulin is indicated below the Western blot images. (**D**) The induction of autophagy assessed by staining for LC3 in CD4^+^ T-cells exposed to EBOV or treated with rapamycin. Left: representative primary flow cytometry data. The gate indicates the position of LC3-positive cells based on the lack of staining with isotype control antibodies. Right: mean values based on triplicate samples from two different donors ± SE. (**E**) Role of GP and TLR4 signaling in induction of autophagy: Jukat cells were incubated with EBOV VLPs, VLPs lacking EBOV GP (VLP-ΔGP) or without VLPs with or without the TLR4 inhibitor CLI-095: representative primary LC3 flow cytometry data (left) and mean LC3 values based on triplicate samples ± SE. The gate indicates the position of LC3-positive cells based on the lack of staining with isotype control antibodies. (**F**) Imaging flow cytometry analysis of autophagy-specific granules of p62-RFP aggregates by an indicator Jurkat cell line. Top: representative images. Bottom: normalized levels of p62-RFP aggregates within the cell. A,B,C: representative of one of two independent experiments. D: representative of one of three independent experiments performed in triplicate samples; histogram is representative of individual samples from one experiment. E: representative images from one of two independent experiments. * P<0.05, ** P<0.01, **** P<0.0001 (Student’s t-test).

The co-localization of virions with markers of autophagosomes suggests that a rapid EBOV-dependent signal may lead to the induction of autophagy. ER stress is often associated with the induction of autophagy [35] and therefore, we sought to determine if high concentrations of EBOV VLPs are capable of inducing a calcium flux following exposure of CD4^+^ T cells. As determined by examining the amount of free versus bound calcium ions, the addition of EBOV VLPs to CD4^+^ T cells resulted in the rapid release of calcium stores (**Fig 5A**). A significant shift in the percentage of cells with free Ca^2+^ ions was observed following stimulation of CD4^+^ T cells with ionomycin or CD3/CD28 beads; the later demonstrates the rapid signal transduction that occurs following receptor-mediated release of Ca^2+^ ions (**Fig 5A**, compare cells in gated population).

Similarly, addition of EBOV VLPs resulted in a significant shift in the percentage of cells with high concentrations of free Ca^2+^ ions, albeit at reduced levels in comparison to both ionomycin and CD3/CD28 stimulated cells. The ratio of bound versus free calcium over time paralleled the end point analysis (**Fig 5A**, bottom panel). The accumulation of Ca^2+^ in the mitochondrial matrix is associated with oxidative stress and has been shown to lead to the transmission and amplification of apoptotic signals [35]. Hence, taken together, the increase in cell death observed following culture of CD4^+^ T cells in the presence of EBOV may at least in part be due to ER-stress following abortive infection.

We next confirmed the induction of ER stress by examining markers associated with the ER-stress response. Specifically, we examined the phosphorylation state of IRE1α, a key regulator of the ER-stress response and cell fate executor [36]. Using a dose dependent assay, we observed a significant increase in the relative levels of phosphorylated IRE1α when CD4^+^ T cells were exposed to increasing MOIs (**Fig 5B**). Assessment of the expression levels of the downstream transcription factor, XBP-1, which is upregulated upon IRE1α-mediated splicing of XBP-1 mRNA [37], revealed a similar dose-dependent effect; the induction of XBP-1 was also observed with gradient-purified EBOV, suggesting that it does not result from any contaminant in the medium (**Fig 5B**). Culturing of T cells with EBOV also resulted in an increase in phospho-eIF2α levels (**Fig 5C**), which suggests that EBOV infection leads to cell stress and is consistent with our data demonstrating the induction of ER-stress response. Based on quantitative analysis, the addition of EBOV to CD4^+^ T cells resulted in a 1.3 ratio of phospho-eIF2α /GAPDH, whereas in mock-treated cells a ratio of 0.6 was observed (**Fig 5C**).

The PP1 inhibitor 1E7-03 was used to determine whether blocking expression of viral proteins *via* reduction in mRNA synthesis would reduce cell stress. 1E7-03 was added one hour prior to the addition of EBOV, which was followed by an overnight incubation at 37ºC. The treatment reduced phospho-eIF2α/tubulin levels from 1.3 in untreated EBOV exposed CD4^+^ T cells to 0.9 in 1E7-03 treated EBOV-exposed cells (**Fig 5C)**. The addition of the serine/threonine specific phosphatase inhibitor, okadaic acid (OA), also reduced phospho-eIF2α/tubulin levels to 0.8, presumably through its ability to block dephosphorylation of VP30, which is required for transcriptional activity of the EBOV polymerase complex. We previously showed that the Src-kinase inhibitor PP2 and the PI3K inhibitor Ly294 reduce binding of EBOV to T cells by reducing basal levels of Tim-1 expression [29]. Treatment of cells with PP2 or Ly294 reduced phospho-eIF2α/tubulin levels to 0.8 and 0.3, respectively (**Fig 5C**).

As we noted that EBOV co-localized with LC3 (**Fig 2A**), a marker of autophagosomes that translocates from the cytoplasm to the forming organelle, and we demonstrated that EBOV induces an ER-stress response early following exposure, we examined the relative levels of activated LC3 (LC3-II) at the autophagosome membrane using a flow cytometry based assay. Twenty-four hours after the addition of EBOV to primary human CD4^+^ T cells at a MOI of 10 PFU/cell, the percentages of cells positive for the activated form of LC3 were greatly increased (**Fig 5D**). The overall increase in staining pattern was consistent with that observed in cells treated with rapamycin, chloroquine and brefeldin A, chemical agents that are associated with the induction of autophagy. Incubation of primary human CD8^+^ T cells with EBOV also resulted in a significant increase in the percentages of LC3^+^ cells (**S5 Fig**). EBOV is a member of the family *Filoviridae* which comprises several viruses which cause a severe human disease and includes Marburg virus (MARV). To test if induction of LC3 is common for filoviruses, we incubated primary CD4^+^ T cells with MARV, at a MOI of 3 or 10 PFU/cell for 24 hours, which again resulted in a significant increase in the percentages of LC3^+^ cells (**S6 Fig**).

Our previous studies demonstrated the role of GP and TLR4 signaling in T lymphocyte death [9]. To check the role of GP and TLR4 signaling in induction of autophagy, Jurkat T cells were exposed to the EBOV VLPs lacking GP [38] in the presence of absence of the TLR4 inhibitor, CLI-095, for 24 hours. Either the lack of GP or treatment with CLI-095 resulted in a significant reduction in expression of LC3 (**Fig 5E**) suggesting the role of GP-mediated TLR4 signaling in the induction of autophagy.

We further assessed whether EBOV induces autophagy by measuring aggregate formation in a Jurkat cell line expressing p62-RFP, which subsequent to the induction of autophagy results in the formation of distinct cytoplasmic aggregates by binding to activated, autophagosome-bound LC3. Cells were exposed to EBOV at a MOI of 3 PFU/ml or treated with rapamycin as positive controls for autophagy, and incubated for 24 hours. Imaging flow cytometry analysis demonstrated a significant increase in the formation of the p62 aggregates by both EBOV and rapamycin (**Fig 5F**). Taken together, our data suggests that EBOV induces autophagy *via* ER-stress dependent pathways.

## Discussion

**Fig 6:**
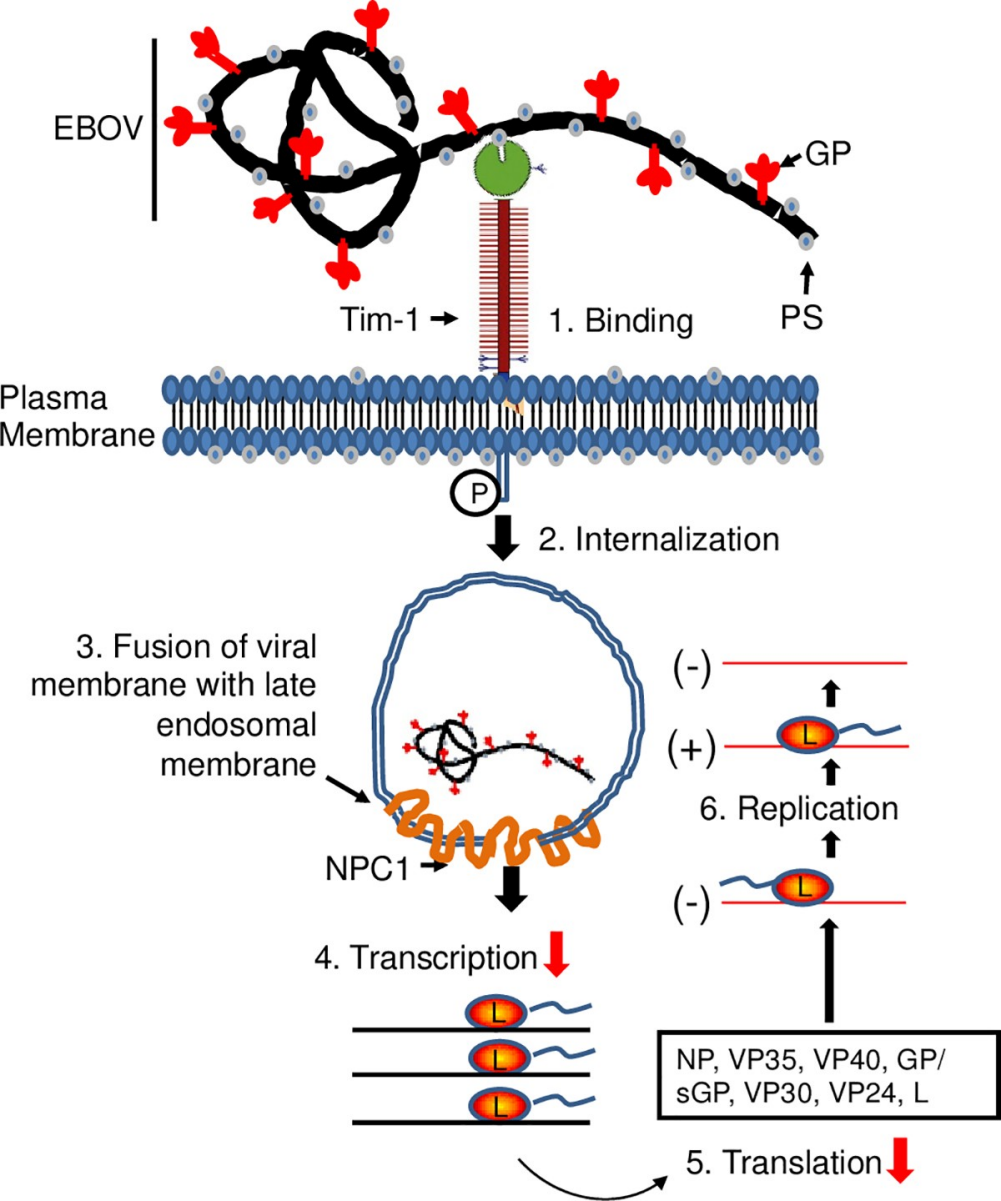
Model of abortive infection in T-cells. (1) Binding of EBOV via PtdSer interaction with Tim-1 on the surface of CD4^+^ T-cells. (2) Internalization of virions into Rab7^+^ and LC3^+^ intracellular vesicles. (3) Fusion, which presumably is occurring due to downstream events. (4–6) Viral RNA and protein synthesis, which are detected at significantly lower levels than those found in permissive cells (red arrows). RNA analysis suggests transcription of viral genes may be blocked in CD4^+^ T-cells.

The results obtained in these studies demonstrate that exposure of T-lymphocytes to EBOV leads to an abortive infection. Together with our previous studies, we demonstrate that EBOV is capable of attaching and entering T-lymphocytes resulting in low level viral polymerase-mediated production of viral RNAs (both gRNA and mRNAs) and proteins. However, no release of infectious virus from EBOV-exposed T lymphocytes was detected. Fusion of permissive cells with the Jurkat CD4^+^ T-cell line impaired productive infection as indicated by the absence of GFP^+^ cells (**Fig 3**). This finding, along with the observed reduction in viral mRNA synthesis strongly suggests the presence of a cellular restriction factor in CD4^+^ T cells that results in an abortive viral infection. Furthermore, the reduced transcription efficiency in the Jurkat T cells and the fusion cell line compared to Huh7 cells (**Fig 3D**) may be explained in part by the greater levels of phosphorylated VP30 which was demonstrated in the two lymphocytic cell lines (**Fig 1I**). Perhaps a restriction factor present in T cells impedes the PP1- or PP2-mediated dephosphorylation of VP30, which is required for the transcriptional activity of the EBOV polymerase complex [30, 39]. Further studies are required to identify the cellular restriction factor(s). A model depicting the potential mechanism of restriction of EBOV replication in T-lymphocytes is provided in **Fig 6**.

Previous findings have demonstrated that Tim-1, which is required for EBOV attachment, and Niemann Pick Protein-1 (NPC-1), which is required for fusion of the EBOV membrane with the cells endosomal membrane enabling entry, are readily detectable in primary CD4^+^ T cells [40–43]. Interestingly, overexpression of Tim-1 on Jurkat T cells was shown to have inconsequential effects as no detectable viral replication was observed [43]. This finding provides additional, indirect evidence of the existence of a cellular restriction factor. As we have shown that viral attachment, entry, RNA synthesis and protein synthesis occur in T cells, our findings strongly suggest that T cells express a cellular restriction factor that inhibits viral replication.

Although Tim-1 is expressed at basal levels on the surface of T cells [40–42], in particular on the surface of memory T cells [29, 44], expression levels are highest on activated Th1 and Th2 subsets, with increased expression occurring for 1 and 3 weeks, respectively [41]. The heightened expression levels may increase the susceptibility of activated T cells to abortive EBOV-infection, which may contribute to the onset of a cytokine storm and subsequent depletion of T-lymphocytes. The depletion of a specific subset that counters pro-inflammatory responses (e.g. Th2 cells) may lead to the exacerbated immunological responses *in vivo* following EBOV infection. Examination of specific T-cell subsets over the course of infection may reveal if certain types of T cells are preferential targets for EBOV-mediated depletion *in vivo*.

Transcription of XBP-1 mRNA increases following IL-2 mediated stimulation, while IRE1α-dependent splicing occurs upon TCR-engagement [45, 46]. As noted in our previous studies, binding of EBOV and subsequent activation of the Tim-1 signaling pathway substitutes for a direct TCR-dependent activating signal, which results in the release of numerous inflammatory mediators including IL-2 [29]. Hence, EBOV binding to Tim-1 on the surface of T cells may trigger IRE1α-dependent splicing of XBP-1. Previous reports have shown that following acute infection, the IRE-1/XBP-1 pathway in effector CD8^+^ T cells is activated [45]. These findings linked the response to a pathogen with the activation of ER stress sensors in the development of end-stage effector cytotoxic T-lymphocytes. It should be noted, however, that the increased phosphorylation of eIF2α in part results from activation of Protein Kinase R by the EBOV double-stranded RNA intermediate, as demonstrated for rotavirus [47]. Thus, the observed ER stress may result directly from the abortive infection and/or indirectly through engagement of plasma membrane associated receptors. Interestingly, a recent study demonstrated that EBOV infection of Huh7 cells, as well as a bat embryonic cell line R06E-J, led to induction of the PPP1R15A protein which is involved in ER stress-induced cell death [48] and references therein. As EBOV readily infects multiple cell types, it will be interesting to test the role of autophagy and ER stress in a wide variety of immune and non-immune cells.

It is feasible that abortive infection of CD4^+^ T cells, which has previously been observed during HIV-infection [49], may partially occur through the phosphatidylserine (PtdSer)-Tim-1 interactions. As noted in the study by Doitsh et al., death of more than 95% of CD4^+^ T cells was attributed to abortive infection. In addition to HIV virion-associated PtdSer having previously been shown to associate with PtdSer-receptors including Tim-1 [50], the HIV envelope protein has also been shown to interact directly with Tim-1 [51].

Interestingly and in support of our findings, a recent study by Menicucci et al. detected a similar viral mRNAs gradient profile in T cells isolated from EBOV-infected macaques [26]. Lastly, we previously demonstrated that exposure of CD4^+^ T cells to EBOV resulted in the induction of both a pro-apoptotic and pro-inflammatory response, which is consistent with the reported effects of HIV abortive infection ultimately attributed to cell death [9, 29, 49].

It remains to be determined if other enveloped viruses that utilize the PtdSer-Tim-1 interaction to gain entry into target cells also result in abortive infection of T cells. It is tempting to speculate that the general incorporation of PtdSer into virions and subsequent interactions with Tim-1 would be a common mechanism utilized by a broad range of viruses; however, the PtdSer-Tim-1 interaction only facilitates attachment and not direct fusion of the viral membrane with endosomal membranes upon entry [43]. Therefore, other cellular factors may be required for efficient fusion of viral membranes within endosomes. The potential general role of PtdSer-Tim-1 interactions in the abortive infection within T cells and subsequent induction of lymphopenia would suggest that blocking EBOV interaction with T cells may prevent the onset of lymphopenia and improve disease outcome.

In conclusion, our study demonstrates for the first time that EBOV causes an abortive infection in T cells, associated with the presence of a restriction factor and leads to induction of ER stress-induced autophagy. Further studies are needed to investigate the role of these processes in EBOV-induced lymphocyte death, immunosuppression and pathogenesis in general.

## Materials and methods

### Viruses and virus-like particles

EBOV strain Mayinga [52] was propagated by 3–4 passages in Vero-E6 cell monolayers. Recombinant EBOV, strain Mayinga, expressing green fluorescent protein (EBOV-GFP) [33] was recovered from transfection of endotoxin-free plasmids encoding viral cDNA as previously described [53] and propagated by 3 passages in Vero-E6 cell monolayers. Viral stocks were quantified by plaque titration in Vero-E6 cell monolayers as previously described [54]. To prepare sucrose-gradient purified EBOV, supernatants of EBOV-infected Vero-E6 cell monolayers were clarified from cell debris by low-speed centrifugation and subjected to ultracentrifugation through 25% sucrose cushion for 2 hours, 175,000 x g, 4°C. Virus pellets were resuspended in STE buffer (0.1 M NaCl, 10 mM Tris-HCl, 1 mM EDTA, pH 8.0) and further purified by ultracentrifugation in 20–60% sucrose gradient for 1.5 hours, 288,000 x g, 4°C. The virus-containing band was collected, virions were resuspended in STE buffer and pelleted by ultracentrifugation for 1 hour, 175,000 x g, 4°C). Purified viral particles were resuspended in a small volume of STE buffer, aliquoted, and kept frozen at -80°C. MARV strain Angola-200501379 isolated during the outbreak in Angola in 2005 [55] was propagated by 4 passages in Vero-E6 cell monolayers. EBOV and MARV stocks were quantified by plaque titration in Vero-E6 cell monolayers as previously described [53, 56].

EBOV VLPs were prepared using the method described previously [57]. Briefly, plasmids encoding NP, GP and codon optimized VP40 were transfected to 293T cell monolayers in 225 cm^2^ cell culture flacks at 120 μg of each plasmid per flask using TransIT-LT1 transfection reagent (Mirus) according manufacturer’s recommendations. To produce VLPs lacking GP, cells were transfected with the NP and VP40 plasmids only. Cells were incubated for thee days and supernatants were harvested. VLPs were purified by sucrose gradient as described above for EBOV virions and quantified by Pierce BCA Protein Assay Kit (ThermoFisher Scientific). The amounts of VLPs which include all three proteins and VLPs lacking GP were normalized by Western blot followed by quantification of NP by densitometry. For the experiments, 2 μg of VLP was added per well of a 96-well deep well plate.

### Primary cells and cell lines

Unidentified buffy coats obtained from the blood of healthy adult donors were used to isolate peripheral blood mononuclear cells (PBMC) by Histopaque (Sigma-Aldrich) gradient as recommended by the manufacturer. Primary human CD4^+^ and CD8^+^ T cells were isolated from PBMCs using negative selection magnetic beads (Miltenyi). Cells were cultured in RPMI 1640 medium (ThermoFisher Scientific) supplemented with 10% HI-FBS (ThermoFisher Scientific), 1% HEPES (Corning) and 2% PenStrep mix (ThermoFisher Scientific). Cytokine free media was used as the culture of T cells with EBOV triggers the production of cytokines, including IL-2, due to superantigen-like activity of the virus [29], and an exogenous supply of IL-2 may trigger cell death.

Human embryonic kidney 293T (293T) and Huh7 cell lines were obtained from the American Type Culture Collection and cultured in Dulbecco’s modified Eagle medium (DMEM) supplemented with 10% heat-inactivated fetal bovine serum (HI-FBS) (ThermoFisher Scientific), 1% HEPES (Corning), 1% nonessential amino acids (Sigma-Aldrich), 1% sodium pyruvate (Sigma-Aldrich), and 2% PenStrep mix (ThermoFisher Scientific). Vero-E6 cells were obtained from the American Type Culture Collection and cultured in Modified Eagle Medium (MEM) supplemented with 10% of HI-FBS, 1%f nonessential amino acids (Sigma-Aldrich), 1% sodium pyruvate (Sigma-Aldrich), and 2% PenStrep mix (ThermoFisher Scientific). The Jurkat and SUP-T1 human T lymphocyte cell lines were obtained from the American Type Culture Collection and cultured in RPMI 1640 (ThermoFisher Scientific) supplemented with 10% HI-FBS (ThermoFisher Scientific), 1% HEPES (Corning) and 2% PenStrep mix (ThermoFisher Scientific).

Generation of the Huh7-Jurkat T-cell fusion cell line was performed by fusing 10x10^6^ DiL-labeled Jurkat T cells with 5x10^6^ DiD-labeled Huh7 cells (both dyes were purchased from ThermoFisher Scientific). The cells were centrifuged together and resuspended gradually in 500 μl of ClonalCell-HY PEG as per manufacturers protocol (Stem Cell Technologies). Fused cells were either used the following day for infection experiments or sorted based on dual staining to make a fusion cell line.

### Analysis of viral RNAs

EBOV-GFP was added to 1x10^6^ isolated primary CD4^+^ T cells at an MOI of 1 PFU/cell and collected on days 1, 2 and 5 post exposure. Cells were pelleted at 400xg for 5 minutes at room temperature and lysed in 1 ml of Trizol reagent (ThermoFisher Scientific). In some experiments, the PP1 inhibitor, 1E7-03, was added 1 hour prior to the addition of EBOV at a concentration of 10 μM, and cells were collected 48 hours post exposure to EBOV. Cell monolayers were rinsed twice with phosphate buffered saline (PBS; Mediatech, Corning) and denatured in 0.8 ml of TRIzol reagent (Ambion, ThermoFisher Scientific). After phase separation with chloroform, the aqueous fraction was mixed with an equal volume of 100% ethanol and subjected to RNA isolation using a Direct-Zol RNA miniprep kit (Zymo Research) according to the manufacturers' recommendations, with on-column DNase 1 treatment. The concentration of the extracted RNA was measured with the NanoDrop 2000 (ThermoFisher Scientific) and adjusted to 100 ng/μl. For strand-specific PCR (Figs 1A, 1G, 1H, 3D and 3E and **S1**A Fig), RT primers that distinguish the EBOV gRNA, cRNA and VP40 mRNA were designed with a unique 18–20 nucleotide tag (highlighted in bold) adjacent to the strand specific sequence [58]. The following RT primers were used–gRNA RT primer: **GGCCGTCATGGTGGCGAAT**ACATTGACCACGCTCATCAGAA; cRNA RT primer: **ATGCCTAGCTGAAGCTAGC**GTGACATATTACTGCCGCAATGAATT; VP40 mRNA RT primer: **CCAGATCGTTCGAGTCGTTTTTTT**TTTTTTCTTAATTAGA. A mixture containing 50–100 ng of RNA and 2 pmol of tagged primer was heated for 5 minutes at 65°C and chilled immediately on ice for 5 minutes. Subsequently, the RT master mix containing 4 μl of 5x first strand buffer, 1 μl 0.1 M dithiothreitol, 1 μl dNTP mix (10 mM each), 1 μl Superscript IV reverse transcriptase (200 U/μl, Invitrogen), 1 μl RNasin Plus RNase inhibitor (40 U/μl, Promega) was added and reactions were incubated at 60°C for 10 minutes followed by heating at 80°C for 10 minutes. All cDNA reactions were diluted 10x with nuclease-free water. The unique tag sequence (bold capital letters) used in the RT primers were used as forward primers in the strand-specific qPCR, whereas both probes and reverse primers were specific to the EBOV genome (capital letters). To increase the fluorescent signal of quantitative PCR reactions, a 12 nucleotide AT-rich flap (shown in lower case letters) was added to the 5’ end of tagged primers [59]. The following primers were used in strand-specific qPCR: gRNA forward: aataaatcataa**GGCCGTCATGGTGGCGAAT**; gRNA; reverse: aataaatcataaGTGCGACCATTTTTCCAGGAATCCT; gRNA probe (FAM fluorophore) -GGCTCGCCAGAATAAACGTTGCA; cRNA forward: aataaatcataa**ATGCCTAGCTGAAGCTAGC**GT; cRNA reverse: aataaatcataaGCCCAGACCTTTCGTTAAAGCT; cRNA probe (FAM fluorophore): GCAACATAATAAACTCTGCACT; mRNA forward: aataaatcataa**CCAGATCGTTCGAGTCG**: VP40 mRNA reverse: aataaatcataaATCCTCAAATTGCCTGCATGCT: VP40 mRNA probe (FAM fluorophore): GGTTGTTCACAATCCAAGTAC. PCR amplification was performed with TaqMan (Applied Biosystems) primer-probe mix using the QX200 Droplet Digital PCR System (BioRad). Four microliters of a 10-fold dilution of the cDNA were added to the ddPCR reaction mixture [10 μl of 2x ddPCR Supermix for Probes (No dUTP), 1 μl of 20x TaqMan custom assay and 5 μl of nuclease-free water]. Reaction mixtures were loaded onto cartridges to create droplets on a QX200 Droplet Generator (BioRad). The droplets were transferred onto 96-well PCR Plates (Eppendorf) and amplified on a C1000 Thermal Cycler with a 96-Deep Well Reaction Module (BioRad). The cycle conditions of qPCR were 95°C 10 min, followed by 39 cycles of 94°C 30 sec and 60°C for 1 min, and a final enzyme deactivation step of 98°C for 10 min. Finally, the PCR plates were loaded onto a Droplet Reader, which quantifies the number of positive and negative droplets in each sample. Analysis was performed using QuantaSoft software to get the final concentrations in each sample.

Quantification of NP mRNA and a housekeeping gene (GAPDH) (Fig 1G) were performed using One-Step RT-ddPCR Advanced kit for probes (BioRad). The following primers were used for the NP gene (2,095 to 2,153 bp): forward primer: GCCACTCACGGACAATGACA; reverse primer: GCATGCGAGGGCTGGTT; probe (FAM): AGAAATGAACCCTCCGGCT. Briefly, 50 pg of RNA was added to 5 μl of Supermix, 2 μl of reverse transcriptase, 1 μl of 300 μM DTT and 1 μl of 20x NP custom TaqMan assay (Life Technologies, ThermoFisher Scientific) for each sample. ddPCR was carried out using the instrumentation and analysis software described above. The cycle conditions were 42°C for 60 min, 95°C for 10 min, followed by 39 cycles of 95°C for 15 sec and 60°C for 1 min, and a final enzyme deactivation step of 98°C for 10 min.

For quantification of individual EBOV mRNAs (Figs 1B–1E and S1B–S1E), the RT primer TTTTTTTTTTTTTTTCTTAAT was used to anneal to part of the unique gene-end sequence from EBOV genome along with poly-A tail of the mRNA. SuperScript IV reverse transcriptase (Thermo Fisher Scientific) was used for first strand cDNA synthesis as per manufacturer’s protocols with some modifications. The following primer-probes were used for quantifying individual mRNAs by ddRT-PCR, as described above (Fig 1B–1E): NP forward (F): GATGAAGGATGAGCCTGTAG: NP reverse (R): CCATGGTGGATATTCCTC; NP probe: ACACGTATCCAGACTCCCT; VP35 F: TCCGCTCTCGAGGTGACA; VP35 R: CAACCTCGATCAATCTTG; VP35 probe: GCGTCCAGTCCCACCATC; VP40 F: TCACCATGGTAATCACACA; VP40 R: TTCTCAATCACAGCTGGAA; VP40 probe: TGTGACACGTGTCATTCTC; VP24 F: ATGAGTCCACACTGAAAG; VP24 R: GATAGCAAGAGAGCTATT; VP24 probe: ATCCTCGACACGAATGCA.

### Deep sequencing

Deep sequencing analysis was performed on isolated CD4^+^ T cells from donors cultured in RPMI 1640 medium in the presence of EBOV at a MOI of 3 PFU/cell. Twenty-four and 96 hours post stimulation, cells were washed with PBS 3 times, lysed in 1 ml of TRIzol (ThermoFisher Scientific) and stored at -80°C. Samples were processed for RNA isolation using the Direct-zol RNA MiniPrep kit (Zymo Research) following manufacturer’s recommendations. RNA quality was assessed on an Agilent 2100 Bioanalyzer using the nanochip format, and only intact RNA was used for constructing the mRNA libraries. Libraries were constructed using the Kapa Stranded mRNA-Seq Kit (Kapa Biosystems) according to the manufacturer’s instructions. Libraries were quality controlled and quantitated using the BioAnalzyer 2100 system and QuBit (Invitrogen). The libraries were clonally amplified and sequenced on an Illumina NextSeq 500 to achieve a target density of approximately 200K-220K clusters/mm^2^ on the flow cell with dual indexed paired end sequencing at a 75 bp length using NextSeq 500 NCS v1.3 software.

Raw reads (75 bp) were mapped using the EBOV reference sequence (NCBI Reference Sequence: NC_002549.1) using BWA mem (version 0.7.15, http://bio-bwa.sourceforge.net/). Visualization of the reads across this reference was accomplished with the Integrative Genomics Viewer (http://software.broadinstitute.org/software/igv/) [60].

### Confocal microscopy

Isolated primary CD4^+^ T cells were grown in suspension at 1x10^6^ cells/well in 12-well plates and loaded on positively charged coverslips (ThermoFisher Scientific) for 2 hours at 37°C. Cells were exposed to EBOV at a MOI of 1 PFU/cell and incubated at 37°C for 2 hours, washed 3 times with PBS, and fixed with 3.2% paraformaldehyde for 15 minutes. Cells were permeabilized with 0.5% Triton X100 (Alfa Aesar) solution in PBS for 15 minutes. Then cells were washed with PBS, incubated with 0.5 M glycine in PBS for 30 minutes at room temperature, and washed 3 times with PBS. Antigen blocking was performed using 5% donkey serum diluted with PBS with 1% bovine serum albumin (BSA) and 0.1% Triton X100 (stain buffer) for 1 hour. Mouse monoclonal antibodies against LC3 and Rab7 (both from Santa Cruz Biotechnology) and rabbit immune serum against EBOV VLP (Integrated BioTherapeutics) were diluted at 1:100 in stain buffer. After 1 hour incubation at room temperature, slides were washed 3 times in staining buffer (0.1% Triton X100 in PBS), incubated for one hour with the mixture of two secondary antibodies: donkey anti-mouse conjugated with Alexa Fluor 488 and donkey anti-rabbit conjugated with Alexa Fluor 647 (both ThermoFisher Scientific) diluted at 1:200 in staining buffer, and washed 3 times in 0.1% Triton X100 in PBS. Next, cells were incubated with 6-diamin-2-phenylindole-dihydrochloride (DAPI) (ThermoFisher Scientific) at the final concentration 1 μg/ml for 2 minutes and washed 3 times in PBS. Slides were then fixed in 4% paraformaldehyde and removed from BSL-4 as described above. Coverslips were mounted onto microscope slides using PermaFluor mounting medium (ThermoFisher Scientific) and analyzed by laser scanning confocal microscopy using Olympus FV1000 confocal microscope. Laser beams with 405 nm wavelengths were used for DAPI excitation, 488 nm for Alexa Fluor 488, and 635 nm for Alexa Fluor 647. Emission filters were 425/25 nm for DAPI, 515/30 nm for Alexa Fluor 488 and 610/50 nm for Alexa Fluor 647 detection, respectively. All images were acquired using a 60x oil objective. In some experiments, EBOV was added to Huh7 and Jurkat T cells for 96 hours prior to processing as indicated above to analyze synthesis and distribution of viral antigens.

### Electron microscopy

Transmission Electron Microscopy (TEM) was performed in the Electron Microscopy Laboratory in the Department of Pathology at the UTMB. Briefly, CD4^+^ T cells were infected at a MOI of 1 PFU/cell for 48 hours, washed and fixed as described below. For ultrastructural analysis of ultrathin sections, infected cells were centrifuged for 5 minutes at 400xg and fixed in a mixture of 2.5% formaldehyde prepared from paraformaldehyde powder, and 0.1% glutaraldehyde in 0.05 M cacodylate buffer pH 7.3 to which 0.01% picric acid and 0.03% CaCl_2_ were added, for 48 hours at room temperature. Fixed cells were washed in 0.1 M cacodylate buffer then post-fixed in 1% OsO_4_ in 0.1 M cacodylate buffer pH 7.3 for 1 hour, washed with distilled water and *en bloc* stained with 2% aqueous uranyl acetate for 20 min at 60°C. The pellets were dehydrated in ethanol, processed through propylene oxide and embedded in Poly/Bed 812 (Polysciences). Ultrathin sections were cut on Leica EM UC7 ultramicrotome (Leica Microsystems), stained with lead citrate and examined in a Philips (FEI) CM-100 transmission electron microscope at 60 kV. Images were acquired with digital camera Orious SC200-1 (Gatan).

Dual immunogold staining was used to detect CD3 and viral antigens. Sections were stained with mouse anti-CD3α monoclonal antibody (1:1,000) (Santa Cruz Biotechnology) and rabbit anti-VLP serum (1:1,000) (IBT BioServices) diluted in PBS containing 0.2% acetylated bovine serum albumin (BSA-c). Secondary antibodies conjugated to gold particles were used to detect primary antibodies. Goat anti-rabbit IgG-conjugated gold particles (15 nm) and goat anti-mouse IgG-conjugated gold particles (40 nm) were purchased from EM Biosciences. Both secondary antibodies were used at a 1:1,000 dilution in PBS containing 0.2% BSA-c and all washes were performed with the same buffer. Primary and secondary antibody staining was performed overnight at 4°C and followed by 3 washes. Post-staining fixation was performed in 2.5% glutaraldehyde in PBS for 2 hours.

### Western blot analysis

CD4^+^ T cells, Jurkat, SUP-T1 or Huh7 cells were exposed to EBOV, lysed in 4x SDS Laemmli Buffer (ThermoFisher Scientific), diluted in RIPA buffer, boiled for 15 minutes and loaded on 4–12% gradient gels (ThermoFisher Scientific). Separated proteins were transferred onto nitrocellulose membranes using the I-Blot2 system (ThermoFisher Scientific) and incubated with primary antibodies overnight at 4°C. For HRP-based blots, all antibodies were diluted in PBST containing 1% dry nonfat milk. For blots which used IRDye 800CW or IRDye 680-labeled secondary antibodies, the primary antibodies were diluted in LI-COR blocking reagent supplemented with 0.1% Tween-20. The following primary antibodies were used: rabbit anti-EBOV VLP serum (1:5,000), rabbit anti-EBOV VP40 (1:1,000), rabbit anti-EBOV NP (1:1,000), rabbit anti-VP30 (1:1,000) (all from Integrated BioTherapeutics), rabbit anti-phosphorylated EBOV VP30 (1:1,000) (described below), IRE1α (1:10,000, Cell Signaling), phospho-IRE1α (1:10,000, Novus Biological), XBP1 (1:10,000, Santa Cruz Biotechnology) and mouse monoclonal GAPDH antibody (1:1,000) (ThermoFisher Scientific). Blots were washed 3 times for 20 minutes each with PBS containing 0.1% Tween-20 (PBST) and incubated for 1–3 hours with secondary antibodies diluted in 1% milk in PBST (anti-mouse and anti-rabbit HRP-labeled antibodies; Santa Cruz Biotechnology) or LI-COR blocking reagent with 0.1% Tween-20 (anti-rabbit IRDye 800CW or anti-mouse IRDye 680-labeled secondary antibodies; LI-COR). HRP-conjugated antibodies were used at 1:5,000 dilution and fluorescent-conjugated antibodies were used 1:20,000 dilution. Following three 20 minutes washes with PBST, the Pierce ECL Plus substrate (ThermoFisher Scientific) was used to detect bands bound by HRP antibodies and the Odyssey LI-COR imager was used to detect fluorescent conjugates. In some experiments, custom siRNAs targeting EBOV NP and VP40 were used to demonstrate *de novo* synthesis. Jurkat cells were transfected with 20 nmol siRNAs using the Neon Transfection system (Invitrogen) as per the manufacturer’s protocol specific for this cell line.

### Generation and validation of antibodies specific to phosphorylated EBOV VP30

Antibodies raised against phosphorylated EBOV VP30 S29-31 (phS29-31) were custom ordered from ProSci. Briefly, two New Zealand white specific pathogen-free rabbits were immunized by subcutaneous route with phosphorylated peptide RAR(p)S(p)S(p)SRENYR in Complete Freund’s Adjuvant and boosted three times with the peptide in Incomplete Freund’s Adjuvant on days 14, 28 and 42. On day 70, immune sera from two rabbits were collected and the IgG was purified by affinity chromatography on columns with bound phosphorylated and non-phosphorylated (RARSSSRENYR) peptides (custom-made by ProSci). The specificity of the antibodies was tested by ELISA with the peptides; the enriched antibodies specific for the phosphorylated peptide did not react with non-phosphorylated peptide.

To demonstrate that the antibodies preferentially recognize the phosphorylated form of EBOV VP30, 6-well plates with 293T cells were transfected with Lipofectamine 3000 (ThermoFisher Scientific) with 2.5 μg of Flag-EBOV VP30-c-Myc expressing plasmid per well and incubated at 37°C for two days. Cells were treated or mock-treated with 100 nM okadaic acid to prevent dephosphorylation of serines and threonines in VP30 [30]. Two hours later, cells were collected, washed with Tris-buffered saline (TBS, 150 mM NaCl, 50 mM Tris-Cl, pH 7.5) and lysed in Tris-HCl buffer (50 mM Tris-Cl, pH 7.5, 0.5 M NaCl) containing 1% NP-40, 0.1% SDS and protease inhibitor cocktail (Sigma Aldrich). Two hundred μg of lysate was immunoprecipitated on protein A/G Plus-agarose (Santa Cruz Biotechnology) with anti-Flag mouse monoclonal antibodies M2 (Sigma-Aldrich) in tris-HCl buffer, pH 7.5, with 0.15 M NaCl and 0.1% NP-40 (Sigma-Aldrich) for 3 hours at 4°C on an orbital shaker. The precipitates were washed with TBST buffer (10 mM tris-HCl, pH 7.5, 0.15 mM NaCl, 0.1% Tween 20) lysed in LDS sample buffer (ThermoFisher Scientific), heated at 70°C for 5 minutes and loaded on 10% NuPAGE bis-tris gel (ThermoFisher Scientific). Separated proteins were blotted onto a PVDF membrane (BioRad) using the Mini Trans-Blot system (BioRad), blocked with 3% BSA in TBST and incubated with the antibodies specific to phosphorylated EBOV VP30 peptide (described in the previous paragraph) at 1:2,000 dilution for 2 hours at room temperature. Blots were washed 3 times for 20 minutes each with TBST on an orbital shaker at room temperature. Next, blots were incubated with secondary anti-rabbit IgG labeled with HRP (Sigma) (1:10,000) for 30 minutes at room temperature and were washed 3 times with TBST for 5 minutes each. Bands were visualized using Pierce ECL Plus substrate (ThermoFisher Scientific) and BioRad ChemiDoc Imaging system. To analyze the immunoprecipitated Flag-VP30-cMyc, the membrane was stripped with 0.1 M glycine, pH 2.5, washed with PBST, blocked with 5% milk in PBST, re-probed with anti-c-Myc monoclonal antibodies (Santa Cruz Biotechnology) at 1:3,000 dilution and secondary anti-mouse IgG labeled with HRP (Sigma) at 1:10,000, and the bands were visualized as above. The Western blotting demonstrated preferential binding of the antibodies to VP30 expressed in the presence of okadaic acid over that expressed in the absence of okadaic acid (S7 Fig).

### Flow cytometry

To analyze virus binding, EBOV was added at a MOI of 3 PFU/cell to 2.5x10^5^ cells in a 96-well plate, and the cells were incubated for 1–2 hours on ice. Cells were washed 3 times with FACS buffer (2% FBS in PBS) and stained for EBOV antigens with rabbit serum raised against EBOV VLPs (IBT BioServices). Cells were washed 3 times as above and incubated with mouse anti-rabbit IgG conjugated with Alexa Fluor 647 (ThermoFisher Scientific). Cells were washed 3 times in FACS buffer, fixed with 4% paraformaldehyde and removed from BSL-4 laboratories for flow cytometry analysis. To analyze Ca^2+^ release, primary CD4^+^ T cells were suspended in PBS at 10^6^ cells/ml in PBS and incubated with 10 μM of Indo-1 Blue dye (BD Biosciences) at 37°C for 60 minutes. Next, cells were centrifuged 5 minutes at 400xg, washed once with PBS and resuspended in PBS. Cells were then cultured with 0.5 μM of ionomycin (Sigma Aldrich) with Dynabeads Human Transactivator CD3/CD28 Beads (ThermoFisher Scientific) according to manufacturer’s recommendations or with EBOV VLPs (IBT BioServices) at 200 μg/ml for 10 min. Ca^2+^ release was analyzed by calculating the ratio of free Ca^2^ (BUV-395)/bound Ca^2^ (Indo-1 Blue) by flow cytometry. To analyze primary human CD4^+^ T cells for Ki-67, cells preincubated with EBOV or medium alone were stained for 30 minutes with rat anti-Ki67 antibody conjugated with PE (clone SolA15, BD Biosciences) in FACS buffer, washed 3 times as above, fixed with 4% paraformaldehyde and analyzed by flow cytometry. To analyze primary CD4^+^ T cells for proliferation, cells were stained with CellTrace-Far-Red (ThermoFisher Scientific) according to manufacturer’s recommendations, incubated with EBOV or medium alone for 3 or 5 days, and analyzed by flow cytometry. Flow cytometry based analysis of autophagy was performed using the FlowCellect Autophagy LC3 Antibody-based Assay kit (Millipore) according to the manufacturer’s recommendations. LIVE/DEAD staining was performed prior to fixation of all samples to enable analysis of live cells. EBOV-infected cells were washed and fixed in 4% paraformaldehyde. Cells were analyzed on a FACSCanto II, LSR Fortessa or BD Accuri C6 Plus flow cytometer (all instruments BD Biosciences). A minimum of 50,000 events were used for analysis using FlowJo software.

### Imaging flow cytometry

To verify the induction of autophagy, indicator p62-RFP Jurkat cells (Millipore) which form distinct aggregates upon the induction of autophagy, were plated at 10^6^ cells/ml and cultured with EBOV at a MOI of 3 PFU/cell for 24 hours or in the presence of 10 μg/ml rapamycin (Sigma-Aldrich) or 10 μg/ml MG132 (Sigma-Aldrich). Next, cells were harvested and stained as described for flow cytometry experiments above. Hoechst staining (ThermoFisher Scientific) was performed to visualize nuclei following fixation of cells with 4% formaldehyde (Polysciences). Labeled cells were analyzed using the Amnis Flowsight Imaging flow cytometer (MilliporeSigma). Graphs represent the normalized analysis of a minimum of 10,000 cells using the IDEAS software to determine the number of aggregates/speckles per cell.

### Statistical analyses

Each independent experiment (donor) was performed in triplicate to rule out experimental bias or random error. Data were analyzed using statistical methods described in figure legends using GraphPad Prizm 6. *P* values of <0.05 were considered statistically significant. Mean and standard error of the mean (SE) was calculated for all graphs.

### BSL-4 work

All work with EBOV was performed within the Galveston National Laboratory biosafety level 4 laboratories. All staff had the appropriate training and permissions and registrations for work with EBOV from the U.S. government. Flow cytometry was performed either in BSL-4 using the Canto-II instrument or infected samples were treated with 4% buffered paraformaldehyde in PBS for 1 hour according to the approved UTMB standard operating procedure protocol and removed from BSL-4 for analysis with the LSR Fortessa flow cytometer available in the UTMB Flow Cytometry Core Facility or BD Accuri C6 Plus flow cytometer available in Dr. Bukreyev’s laboratory. Cell lysates for Western blot analysis were prepared by lysis of cells with 4x SDS-Laemmli buffer, followed by incubation at 95°C for 15 minutes, vortexing, and removal from BSL4 laboratories. Cells for confocal microscopy were placed on slides, stained, fixed in 4% paraformaldehyde for 24 hours, which was replaced with a fresh solution, incubated for additional 48 hours, and taken out of BSL-4.

### Ethics statement

Unidentified buffy coats were obtained from the blood of healthy adult donors according to a clinical protocol approved by the University of Texas Medical Branch at Galveston (UTMB) Institutional Review Board were provided by the UTMB Blood Bank.

## Supporting information

S1 Fig Abortive EBOV infection in Jurkat cells. The number of viral genomic RNA (**A**) and specific viral mRNAs (**B-E**) copies/ng in CD4^+^ T-cells exposed to EBOV for 1, 2 and 5 days, determined by DDRT-PCR with background signals in mock-infected cells subtracted.
(PDF)

S2 Fig Dual immuno-gold labeling TEM of CD4^+^ T-cells mock-exposed to EBOV: immunostained for CD3 with ~40 nm gold particles (black arrows) and for EBOV antigens with ~15 nm gold particles (not present). TEM of CD4^+^ T-cells exposed to EBOV is shown in Fig 2B.
(PDF)

S3 Fig Confocal microscopy analysis of viral antigens. Detection of EBOV antigens in Huh7 (**A**) and Jurkat (**B**) cells following exposure to the virus. Cells are stained with EBOV-specific antibodies (green) and nuclei are stained with DAPI (blue). White arrows indicate EBOV inclusion bodies.
(PDF)

S4 Fig CD4+ T-cells do not produce infectious virus. (**A, B**) Flow cytometry analysis of GFP^+^ Huh7 (A) and Jurkat (B) cells exposed to EBOV-GFP at MOI of 3 PFU/cell at 48 h post infection. (**C, D**) Flow cytometry analysis of Vero-E6 cells cultured with 50 μl of cell-free supernatants collected from the EBOV-exposed Huh7 (C) or Jurkat (D) cells. Representative dot plots with indicated percentages of the gated populations and histograms. Two independent experiments in triplicates were performed.
(PDF)

S5 Fig Incubation of primary human CD8^+^ T-cells with EBOV induced expression of LC3. CD8^+^ T cells from donor blood were incubated with EBOV at a MOI of 3 for 24 hours, and expression of LC3 was analyzed by flow cytometry. Left: representative primary data. The gate indicates the position of LC3-positive cells based on the lack of staining with isotype control antibodies. Right, percentages of LC3^+^ cells based on triplicate samples analyzed. Data for one of two donors analyzed shown. *** P<0.001 (Student’s t-test).
(PDF)

S6 Fig Incubation of primary human CD4^+^ T-cells with MARV induced expression of LC3. CD4^+^ T cells from donor blood were incubated with MARV at MOI of 3 or 10 PFU/cell for 24 hours, and induction of autophagy assessed by staining for LC3 was analyzed by flow cytometry. Left: representative primary data. The gate indicates the position of LC3-positive cells based on the lack of staining with isotype control antibodies. Right, percentages of LC3^+^ cells based on triplicate samples analyzed. Data for one of two donors analyzed shown. *P<0.05, ** P<0.01, (Student’s t-test).
(PDF)

S7 Fig Characterization of affinity purified immunoglobulins raised against the phosphorylated VP30 peptide. To characterize affinity purified antibodies, 293T cells were transfected with a plasmid expressing EBOV VP30 fused to FLAG and c-myc. Cells were incubated in the absence or presence of 100 nM of okadaic acid, which inhibits PP1 and PP2A, and thereby increases phosphorylation of serines 29, 30 and 31 of EBOV VP30 protein. The protein was immunoprecipitated with anti-FLAG antibodies and the bands were visualized by Western blot with antibodies raised against the EBOV VP30 phosphorylated peptide RAR(p)S(p)S(p)SRENYR (a-phS29-31, the top blot) or with a monoclonal antibody specific for c-Myc (the bottom blot).
(PDF)
