# Supplementary material for: Ebola virus-mediated T-lymphocyte depletion is the result of an abortive infection
Source: PLoS Pathog. 2019 Oct 24;15(10):e1008068. doi: 10.1371/journal.ppat.1008068 (PMC6812753; doi:10.1371/journal.ppat.1008068)
Supplement: S1 Fig — The number of viral genomic RNA (A) and specific viral mRNAs (B-E) copies/ng in CD4+ T-cells exposed to EBOV for 1, 2 and 5 days, determined by DDRT-PCR with background signals in mock-infected cells subtracted. (PDF) [file ppat.1008068.s001.pdf]

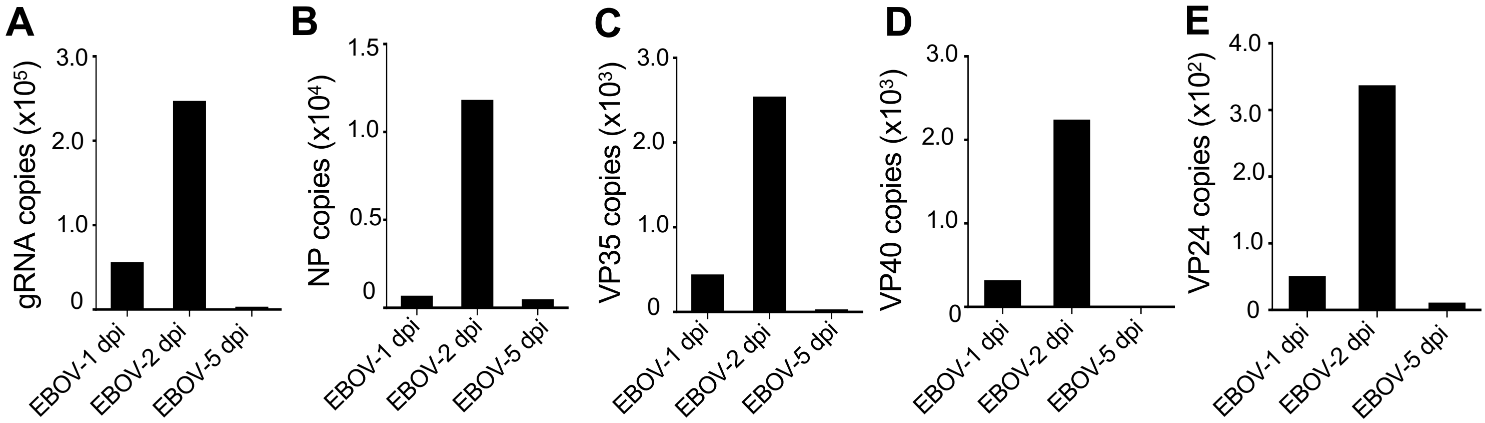

Supplementary Figure 1. Abortive EBOV infection in Jurkat cells. The number of viral genomic RNA (**A**) and specific viral mRNAs (**B-E**) copies/ng in $CD4^+$ T-cells exposed to EBOV for 1, 2 and 5 days, determined by DDRT-PCR with background signals in mock-infected cells subtracted.
