# Supplementary material for: Ebola virus-mediated T-lymphocyte depletion is the result of an abortive infection
Source: PLoS Pathog. 2019 Oct 24;15(10):e1008068. doi: 10.1371/journal.ppat.1008068 (PMC6812753; doi:10.1371/journal.ppat.1008068)
Supplement: S2 Fig — TEM of CD4+ T-cells exposed to EBOV is shown in Fig 2B. (PDF) [file ppat.1008068.s002.pdf]

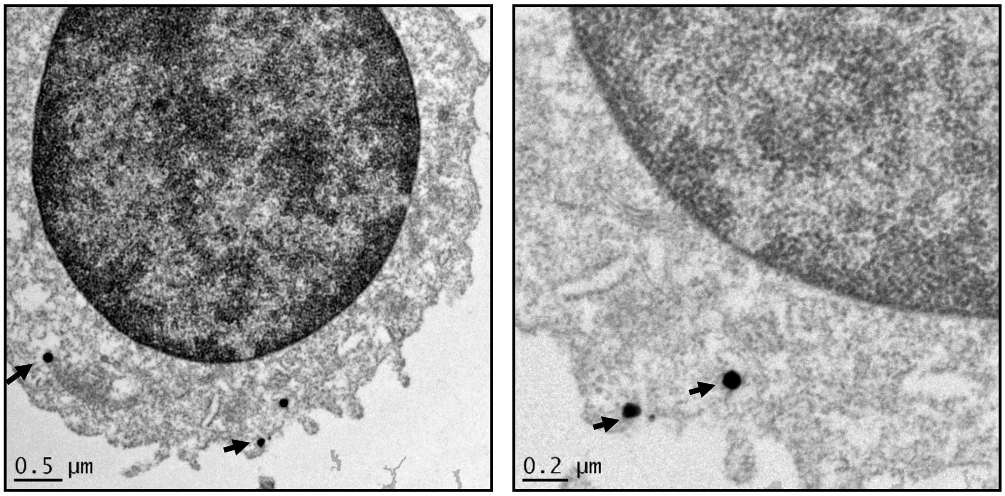

Supplementary Figure 2. Dual immuno-gold labeling TEM of CD4^{+} T-cells mock-exposed to EBOV: immunostained for CD3 with ~40 nm gold particles (black arrows) and for EBOV antigens with ~15 nm gold particles (not present). TEM of CD4^{+} T-cells exposed to EBOV is shown in Figure 2B.
