# Supplementary material for: Ebola virus-mediated T-lymphocyte depletion is the result of an abortive infection
Source: PLoS Pathog. 2019 Oct 24;15(10):e1008068. doi: 10.1371/journal.ppat.1008068 (PMC6812753; doi:10.1371/journal.ppat.1008068)
Supplement: S3 Fig — Detection of EBOV antigens in Huh7 (A) and Jurkat (B) cells following exposure to the virus. Cells are stained with EBOV-specific antibodies (green) and nuclei are stained with DAPI (blue). White arrows indicate EBOV inclusion bodies. (PDF) [file ppat.1008068.s003.pdf]

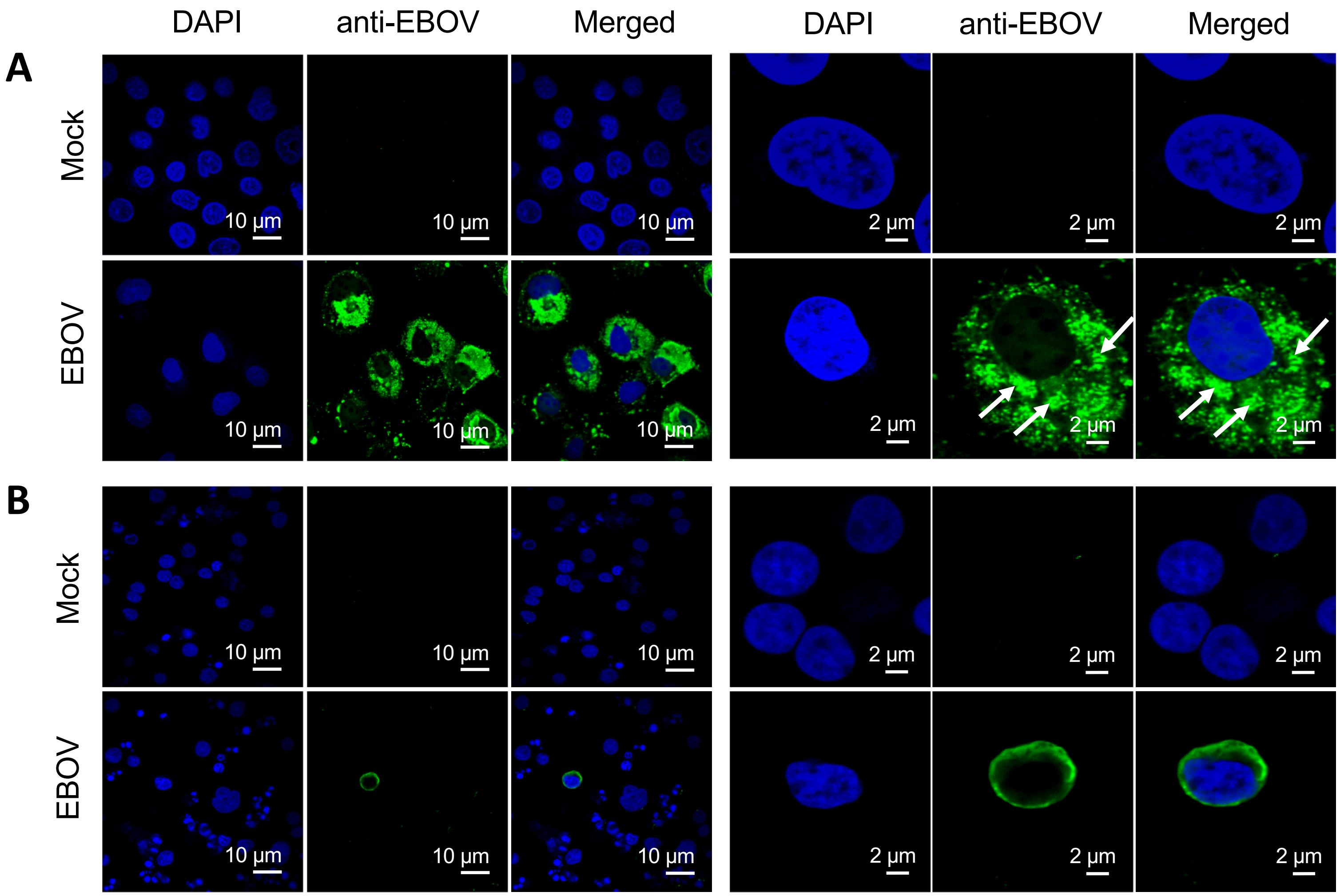

Supplementary Figure 3. Confocal microscopy analysis of viral antigens. Detection of EBOV antigens in Huh7 (**A**) and Jurkat (**B**) cells following exposure to the virus. Cells are stained with EBOV-specific antibodies (green) and nuclei are stained with DAPI (blue). White arrows indicate EBOV inclusion bodies.
