# Supplementary material for: Ebola virus-mediated T-lymphocyte depletion is the result of an abortive infection
Source: PLoS Pathog. 2019 Oct 24;15(10):e1008068. doi: 10.1371/journal.ppat.1008068 (PMC6812753; doi:10.1371/journal.ppat.1008068)
Supplement: S4 Fig — (A, B) Flow cytometry analysis of GFP+ Huh7 (A) and Jurkat (B) cells exposed to EBOV-GFP at MOI of 3 PFU/cell at 48 h post infection. (C, D) Flow cytometry analysis of Vero-E6 cells cultured with 50 μl of cell-free supernatants collected from the EBOV-exposed Huh7 (C) or Jurkat (D) cells. Representative dot plots with indicated percentages of the gated populations and histograms. Two independent experiments in triplicates were performed. (PDF) [file ppat.1008068.s004.pdf]

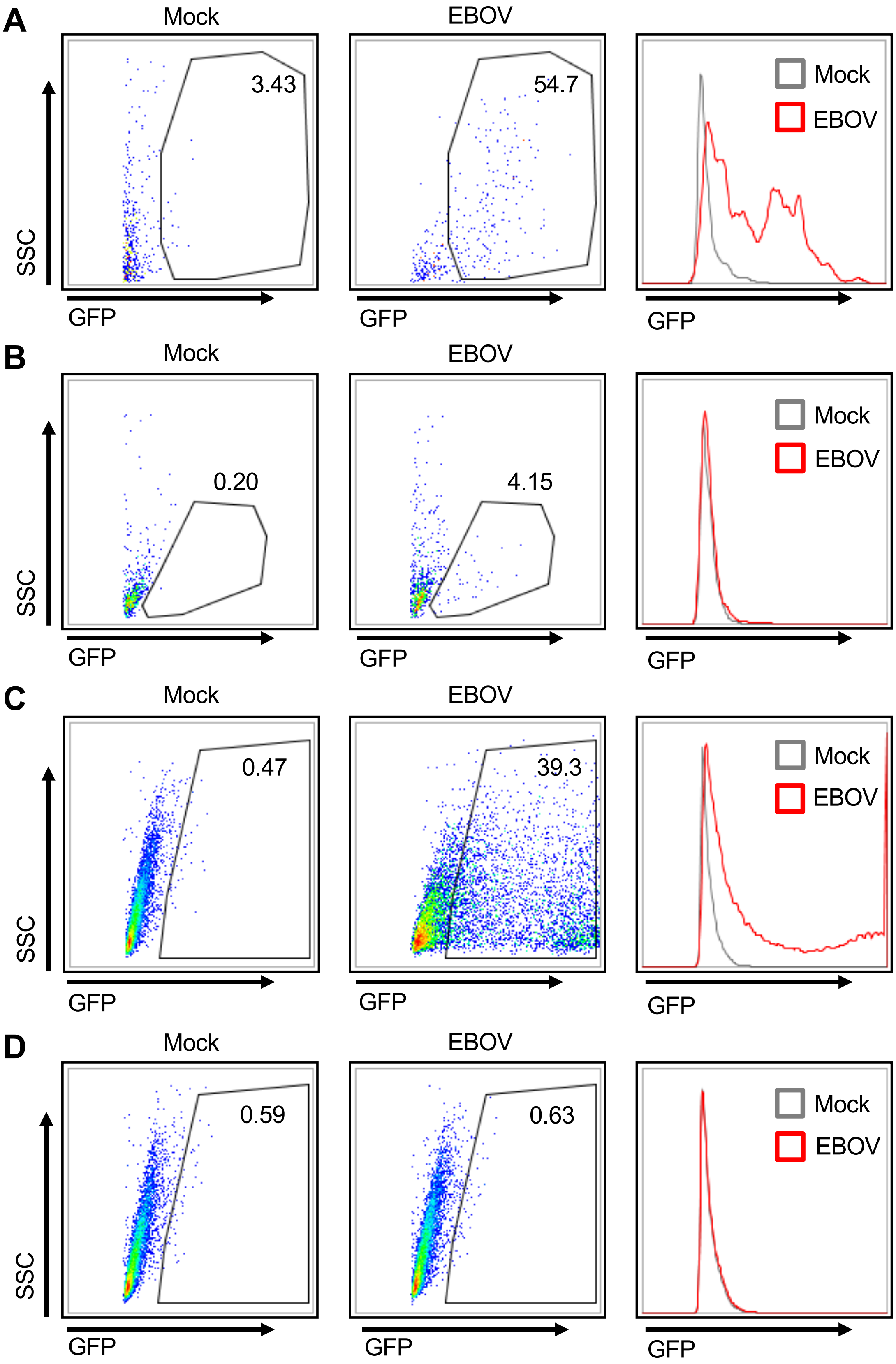

Supplementary Figure 4. CD4⁺ T-cells do not produce infectious virus. (**A, B**) Flow cytometry analysis of GFP⁺ Huh7 (A) and Jurkat (B) cells exposed to EBOV-GFP at MOI of 3 PFU/cell at 48 h post infection. (**C, D**) Flow cytometry analysis of Vero-E6 cells cultured with 50 μl of cell-free supernatants collected from the EBOV-exposed Huh7 (C) or Jurkat (D) cells. Representative dot plots with indicated percentages of the gated populations and histograms. Two independent experiments in triplicates were performed.
