# Supplementary material for: Ebola virus-mediated T-lymphocyte depletion is the result of an abortive infection
Source: PLoS Pathog. 2019 Oct 24;15(10):e1008068. doi: 10.1371/journal.ppat.1008068 (PMC6812753; doi:10.1371/journal.ppat.1008068)
Supplement: S5 Fig — CD8+ T cells from donor blood were incubated with EBOV at a MOI of 3 for 24 hours, and expression of LC3 was analyzed by flow cytometry. Left: representative primary data. The gate indicates the position of LC3-positive cells based on the lack of staining with isotype control antibodies. Right, percentages of LC3+ cells based on triplicate samples analyzed. Data for one of two donors analyzed shown. *** P<0.001 (Student’s t-test). (PDF) [file ppat.1008068.s005.pdf]

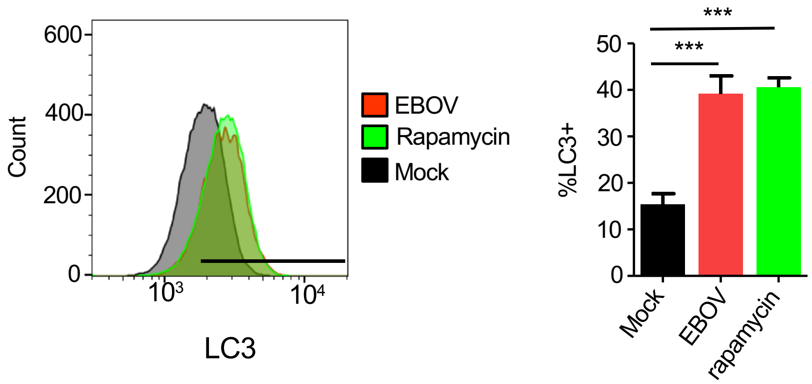

Supplementary Figure 5. Incubation of primary human CD8⁺ T-cells with EBOV induced expression of LC3. CD8⁺ T cells from donor blood were incubated with EBOV at a MOI of 3 for 24 hours, and expression of LC3 was analyzed by flow cytometry. Left: representative primary data. The gate indicates the position of LC3-positive cells based on the lack of staining with isotype control antibodies. Right, percentages of LC3⁺ cells based on triplicate samples analyzed. Data for one of two donors analyzed shown. *** P<0.001 (Student's t-test).
