# Supplementary material for: Ebola virus-mediated T-lymphocyte depletion is the result of an abortive infection
Source: PLoS Pathog. 2019 Oct 24;15(10):e1008068. doi: 10.1371/journal.ppat.1008068 (PMC6812753; doi:10.1371/journal.ppat.1008068)
Supplement: S7 Fig — To characterize affinity purified antibodies, 293T cells were transfected with a plasmid expressing EBOV VP30 fused to FLAG and c-myc. Cells were incubated in the absence or presence of 100 nM of okadaic acid, which inhibits PP1 and PP2A, and thereby increases phosphorylation of serines 29, 30 and 31 of EBOV VP30 protein. The protein was immunoprecipitated with anti-FLAG antibodies and the bands were visualized by Western blot with antibodies raised against the EBOV VP30 phosphorylated peptide RAR(p)S(p)S(p)SRENYR (a-phS29-31, the top blot) or with a monoclonal antibody specific for c-Myc (the bottom blot). (PDF) [file ppat.1008068.s007.pdf]

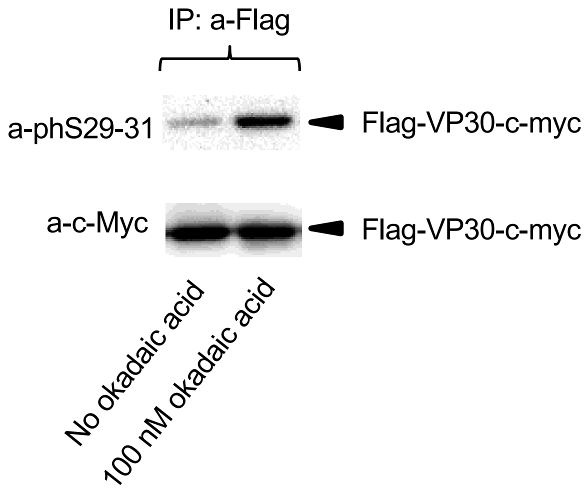

Supplementary Figure 7. Characterization of affinity purified immunoglobulins raised against the phosphorylated VP30 peptide. To characterize affinity purified antibodies, 293T cells were transfected with a plasmid expressing EBOV VP30 fused to FLAG and c-myc. Cells were incubated in the absence or presence of 100 nM of okadaic acid, which inhibits PP1 and PP2A, and thereby increases phosphorylation of serines 29, 30 and 31 of EBOV VP30 protein. The protein was immunoprecipitated with anti-FLAG antibodies and the bands were visualized by Western blot with antibodies raised against the EBOV VP30 phosphorylated peptide RAR(p)S(p)S(p)SRENYR (a-phS29-31, the top blot) or with a monoclonal antibody specific for c-Myc (the bottom blot).
